# Multimodal scanning of genetic variants with base and prime editing

**DOI:** 10.1038/s41587-024-02439-1

**Published:** 2024-11-12

**Authors:** Olivier Belli, Kyriaki Karava, Rick Farouni, Randall J. Platt

**Affiliations:** 1 Department of Biosystems Science and Engineering, ETH Zurich, Basel, Switzerland; 2 Basel Research Centre for Child Health, Basel, Switzerland; 3 Department of Chemistry, University of Basel, Basel, Switzerland; 4 NCCR Molecular Systems Engineering, Basel, Switzerland

**Keywords:** Mutagenesis, Genetic engineering, Cancer genomics

## Abstract

Mutational scanning connects genetic variants to phenotype, enabling the interrogation of protein functions, interactions and variant pathogenicity. However, current methodologies cannot efficiently engineer customizable sets of diverse genetic variants in endogenous loci across cellular contexts in high throughput. Here, we combine cytosine and adenine base editors and a prime editor to assess the pathogenicity of a broad spectrum of variants in the epithelial growth factor receptor gene (*EGFR*). Using pooled base editing and prime editing guide RNA libraries, we install tens of thousands of variants spanning the full coding sequence of *EGFR* in multiple cell lines and assess the role of these variants in tumorigenesis and resistance to tyrosine kinase inhibitors. Our *EGFR* variant scan identifies important hits, supporting the robustness of the approach and revealing underappreciated routes to EGFR activation and drug response. We anticipate that multimodal precision mutational scanning can be applied broadly to characterize genetic variation in any genetic element of interest at high and single-nucleotide resolution.

## Abstract (web summary)

Thousands of rare oncogene variants are evaluated using multimodal gene editing screens.

## Main

Since the publication of the first human genome, deep sequencing technologies have allowed for the identification of many disease-associated genetic variants^1–3^. However, the accurate classification of these variants and their clinical impacts continues to fall behind. Indeed, the fraction of variants listed in ClinVar as variants of uncertain significance (VUS) has been steadily increasing over time to currently represent 47% of the database^4–6^. This not only represents a diagnostic challenge for physicians but also results in patients with noncanonical mutations receiving inequitable access to new medicines. In this context, the development of new technologies for comprehensive and accurate classification of VUS is needed.

A notable example of how VUS can affect clinical practice is underscored by the epithelial growth factor receptor gene (*EGFR*), a member of the receptor tyrosine kinase (RTK) family and known oncogene, common in non-small cell lung cancers (NSCLC) among others^7–9^. Approximately 50% of *EGFR* variants in the ClinVar database are classified as VUS^4^, which can drastically impact clinical treatment and the progression of oncogenesis and drug resistance. To treat cancers driven by EGFR and other RTK family members, different classes of tyrosine kinase inhibitors (TKIs) have been developed. Gefitinib was the first approved, which initially showed positive results, but drug resistance is prevalent because of the emergence of genetic variants such as Thr790Met that impair drug binding^10–12^. Newer generations of TKIs such as osimertinib were developed to specifically counteract Thr790Met^13,14^ but eventually suffer from drug resistance because of a different spectrum of genetic variants^15,16^. Therapeutic decisions are further complicated by the fact that newly developed TKIs are generally only tested against canonical *EGFR* mutations^17^. Indeed, recruiting participants harboring uncommon *EGFR* variants in clinical trials poses a notable challenge^18^, although they have been shown to represent 11% of people with NSCLC^19,20^ and to generally suffer from poorer clinical outcomes^20^. The situation with *EGFR* and TKIs is just a representative example of the challenges faced for a broad range of cancers and therapeutics. As the cancer therapeutic toolbox rapidly expands to overcome resistance, it is essential to establish efficient variant profiling pipelines to allow for the assessment of pathogenicity and drug prioritization^21^.

Several multiplex assays of variant effect (MAVEs) have been developed to assess the functional relevance of mutations of a gene of interest in high throughput^22^. Among these, saturation mutagenesis screens rely on the delivery of a protein-coding gene variant library in reporter cells before measuring the phenotype linked to each variant^20,23–25^. Although this approach presents the clear advantage of systematically assessing every possible substitution in a protein sequence, it fails to replicate the genomic context and endogenous expression levels of the considered gene. More recently, clustered regularly interspaced short palindromic repeats (CRISPR)-based screening approaches were developed and applied in MAVEs. For instance, homology-directed repair (HDR)-based screens using Cas9, a single guide RNA (sgRNA) and a donor template library can theoretically introduce any type of short mutation. However, their scale is limited by the fact that each DNA template in an HDR donor library must be matched to an sgRNA delivered separately, thus preventing the use of this approach to cover full mammalian genes^26^. This limitation was addressed by the development of base editors, which allow for the scarless conversion of single bases using an sgRNA and a Cas enzyme fused to a deaminase domain^27–30^. This technology has been applied in a screening context and was capable of faithfully identifying pathogenic variants in cellular assays^31–34^. However, base editing cannot induce all possible base conversions; therefore, only a subset of all possible ClinVar variants can be assessed. Furthermore, base editors introduce bystander edits within their editing window, leading to a disconnect between the phenotype and inferred genotype. Prime editing overcomes these limitations by introducing any possible small mutation through the action of a viral reverse transcriptase and a prime editing guide RNA (pegRNA)^35–37^, yielding a system that holds remarkable potential in the high-throughput functional characterization of VUS^38–40^.

Here, we leverage base editing and prime editing technologies to establish complementary MAVEs, which we apply to *EGFR* in human cancer and noncancer cells. We first show that full-length *EGFR* mutational scanning using cytidine and adenine base editors (CBEs and ABEs, respectively) captures both known and previously uncharacterized EGFR-activating mutations in a nontumorigenic cell line. We then expand this approach to compare drug resistance profiles in cell lines harboring wild-type or constitutively active EGFR and uncover genetic background-specific mutations conferring resistance to first-generation and third-generation TKIs. Lastly, we demonstrate a prime editing screening approach introducing thousands of patient-derived mutations and capture EGFR-activating variants that could not be modeled by base editors, including sequence insertions. Overall, our results indicate that, in addition to canonical resistance emerging through mutations impacting residues in the EGFR TKI-interacting domain, unexpected alternative mechanisms of resistance likely mediated through alteration in EGFR autoregulation are possible and drug dependent. These results establish multimodal base editing and prime editing scanning as a robust and high-throughput framework for linking variant to phenotype, using *EGFR* as a proof of concept. We envision that multimodal precision gene editing screens could be used to evaluate thousands of rare variants in parallel in clinically relevant genomic contexts, which could allow for quick drug prioritization and the establishment of patient-specific treatment courses.

## Results

### Pathogenic *EGFR* variant modeling in MCF10A cells

Assessing the pathogenicity of *EGFR* variants in high throughput requires experimental models enabling the linkage of genotype to phenotype. We first set out to assess the impact of primary *EGFR* mutations in MCF10A cells, an epithelial cell line isolated from a benign mammary tumor. These cells represent an attractive model because they harbor wild-type EGFR and rely on its signaling for growth. Indeed, MCF10A cells overexpressing hyperactive EGFR variants found in patients have been shown to proliferate in the absence of EGF supplementation while cells expressing wild-type EGFR remain quiescent^41,42^. We, thus, envisioned that introducing EGFR-activating mutations in the genome of MCF10A cells could make edited cells EGF independent and that these cells would outcompete unedited cells when deprived of EGF, enabling us to connect genotype to phenotype in a pooled screen.

**Extended Data Fig. 1:**
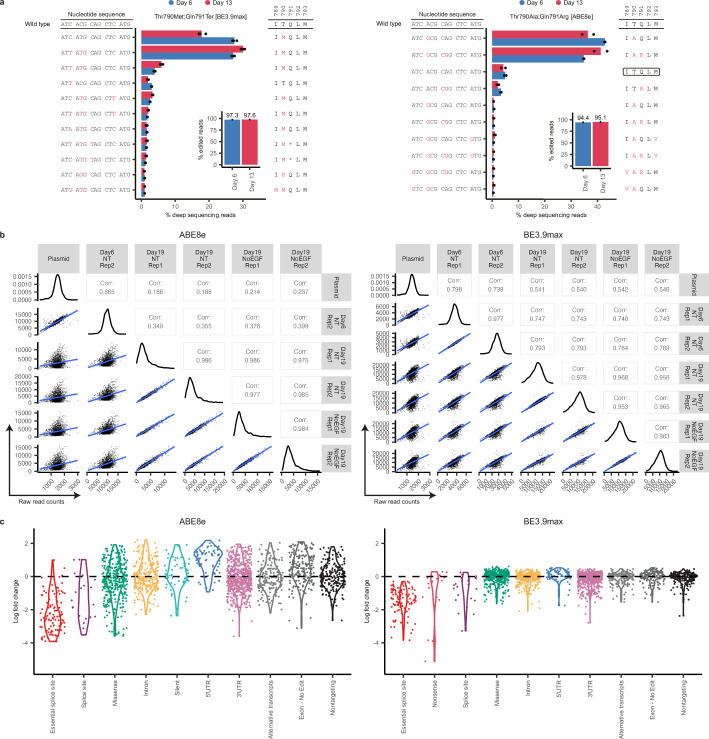
Evaluation of ABE8e and BE3.9max activities in MCF10A cells. (**a**) Global editing efficiencies and deep sequencing read distributions from base editing experiments in MCF10A cells. The same sgRNA targeting Thr790 was delivered with the BE3.9max (*n* = 3, left) or ABE8e (*n* = 2, right) base editors. Target sequencing was performed on day 6 and 13 after lentiviral infection. The frequencies of the ten most prevalent reads are shown for each sample. Edited nucleotides and amino acids are shown in red, black boxes show wild-type sequences. (**b**) sgRNA library distributions and sample correlations for the ABE8e and BE3.9max EGFR activation screen in MCF10A cells. Raw read counts and Pearson correlation coefficients are shown for all samples. (**c**) Impact of individual sgRNAs on cell viability in the base editing EGFR activation screen in MCF10A cells. LFCs of individual sgRNAs between plasmid and day 19 (nontreated) are shown for each target category or predicted mutation type.

First, we evaluated base editing efficiency in MCF10A cells by lentiviral delivery of the CBE BE3.9max or the ABE ABE8e along with an sgRNA predicted to introduce a mutation impacting the ‘gatekeeper’ residue Thr790. Deep sequencing of the target exon revealed efficient editing at the target site with 97% or 95% of reads containing at least one base change 6 days after infection for BE3.9max or ABE8e, respectively, indicating high editing efficiency for both base editors (Extended Data Fig. 1a). Although the total fraction of edited alleles remained stable over time, the relative proportions of editing products varied between day 6 and day 13, with alleles containing two or three edits being enriched as opposed to single edits. This is likely explained by sequential base editing of less preferred collateral bases throughout time and as the preferred base conversion has already been installed on most alleles. This potentially complicates the accurate prediction of variant effects but can also serve to broaden the mutational spectrum that can be assessed in a single experiment.

### Base editing scanning identifies loss-of-function (LOF) variants

**Fig. 1:**
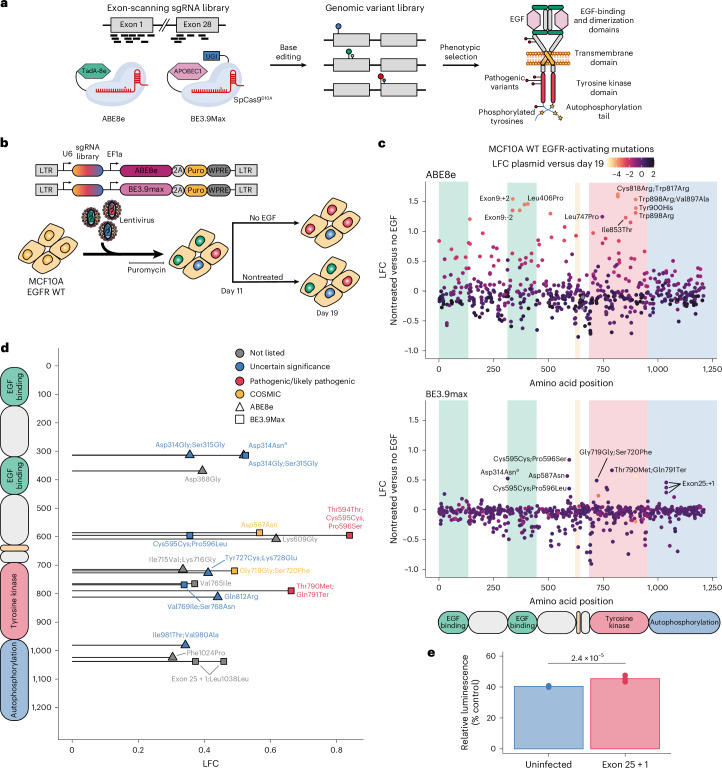
Base editing scanning screens identify EGFR-activating mutations in MCF10A cells. **a**, Schematic of the base editing mutational scanning approach applied to *EGFR*. UGI, uracil DNA glycosylase inhibitor. **b**, Overview of the base editing constructs and workflow to identify activating *EGFR* mutations. All-in-one base editing libraries packaged in lentivirus were delivered to MCF10A cells in duplicate. Cells were split into nontreated and EGF-deprived treatment arms on day 11 and harvested on day 19. WT, wild type; LTR, long terminal repeat; WPRE, Woodchuck hepatitis virus (WHV) posttranscriptional regulatory element. **c**, Scatter plot showing the LFCs of individual sgRNAs along the EGFR protein between nontreated and EGF-deprived cells when using ABE8e (top) or BE3.9max (bottom). LFCs between the plasmid library and nontreated cells on day 19 are shown as color gradients. Background colors represent EGFR domains. **d**, Diagram showing combined ABE8e and BE3.9max screen hits along the EGFR protein. Hits are defined as sgRNAs with LFCs > −0.6 between plasmid and day 19 and LFCs > 0.3 between nontreated and EGF-deprived cells. Shapes and colors represent the corresponding base editor and classification of the predicted amino acid changes, respectively. The COSMIC category is only shown if the mutation is not listed in ClinVar. **e**, Viability assay for individual sgRNA validation. Viability comparison between uninfected cells and cells infected with an sgRNA, in three biological replicates, introducing the Leu1038Leu;exon 25 + 1 [BE3.9max] mutation as measured by the CellTiter-Glo 2.0 assay after 5 days of EGF deprivation. Relative luminescence intensities compared to nontreated cells seeded at the same time are shown with unpaired two-sided *t*-test *P* values (*n* = 3). Mean uninfected, 40.43%; mean exon 25 + 1, 45.40%. ^a^Validation of the Asp314Asn [BE3.9max] sgRNA showed that the main variant conferring EGF-independent growth was Glu317Lys (Extended Data Fig. 2d). Source data

To evaluate the potential of CBEs and ABEs applied in a MAVE context, we set out to apply the tools to interrogate a spectrum of *EGFR* mutations. We designed a base editing variant-scanning library composed of 1,496 unique sgRNAs targeting all *EGFR* exons (Fig. 1a). We also included 200 sgRNAs targeting an *EGFR* intronic region, 206 nontargeting sgRNAs as negative controls and 103 sgRNAs targeting splice sites of essential genes as positive controls. To explore a broad range of genetic variants accessible by base editing, we cloned this library under the control of a U6 promoter in two different lentiviral backbones expressing either ABE8e or BE3.9max. The two resulting all-in-one base editing sgRNA libraries were quantified by deep sequencing and showed no dropouts and a narrow sgRNA distribution with skew ratios of 1.6 (Extended Data Fig. 1b).

We then delivered both libraries to MCF10A cells by lentiviral infection (Fig. 1b). Each infection was performed in duplicate at a low multiplicity of infection (MOI) with an average coverage of 500 cells per sgRNA. After puromycin selection of transduced cells and EGF deprivation, genomic DNA was extracted and sgRNA libraries were prepared and sequenced while ensuring appropriate coverage and depth. The sgRNA counts were then quantified from sequencing data using a custom script and normalized to nontargeting sgRNAs with MAGeCK^43^. We then confirmed library coverage and replicate correlation at every time point (Extended Data Fig. 1b), overall indicating a robust dataset warranting further analysis.

**Extended Data Fig. 2:**
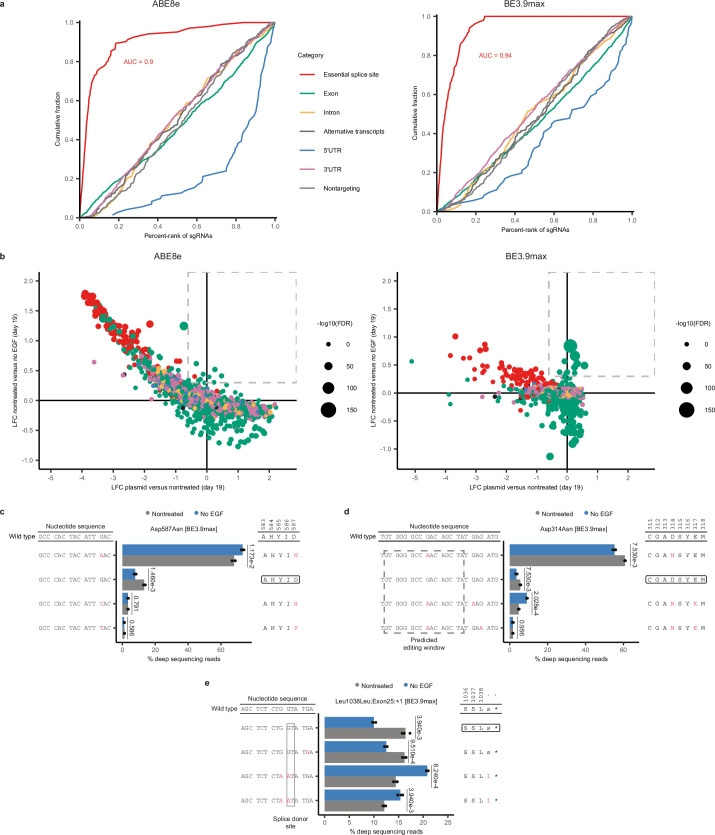
sgRNA impacts on viability and EGF-independent growth in the base editing EGFR activation screen in MCF10A cells. (**a**) Receiver operating characteristic (ROC) curves for each target category or predicted mutation type when comparing plasmid and day 19 (nontreated). The AUC for essential splice site sgRNAs are shown in red. (**b**) Scatter plot comparing the individual sgRNA LFCs between plasmid and day 19 and between nontreated and EGF-deprived samples. Negative-log FDR values for EGFR activating sgRNAs are shown. Dashed squares represent the hit inclusion criteria: LFC (plasmid versus day 19) > -0.6 and LFC (nontreated versus EGF-deprived) > 0.3. (**c**–**e**) Deep sequencing data for individual sgRNA validation in MCF10A cells. Cells were infected with the indicated sgRNA and base editor pairs in three biological replicates (*n* = 3) and later split into control and treatment arms deprived of EGF. The percentages of reads corresponding to the four most represented alleles at each target site are shown for both nontreated and EGF-deprived samples. Nucleotide sequences represent the coding DNA strand and black squares denote wild-type *EGFR* sequences. Holm-adjusted *P* values are shown for two-sided *t*-tests.

We started our analysis by comparing the relative abundances of sgRNAs between the plasmid library and late (day 19) time point, which should reveal sgRNAs that impact MCF10A viability. As expected, we observed no change in the nontargeting negative control sgRNAs but a strong depletion of gene editing positive control sgRNAs targeting splice sites of essential genes with both base editors (Extended Data Fig. 1c). ABE8e and BE3.9max screens distinguished between essential splice site controls and other sgRNAs with areas under the curve (AUCs) of 0.9 and 0.94, respectively, confirming the ability of our pooled library to efficiently introduce edits and elicit measurable phenotypes (Extended Data Fig. 2a).

Encouragingly, multiple sgRNAs predicted to introduce splice site, nonsense, and missense mutations in *EGFR* were depleted on day 19 with both base editors (Extended Data Fig. 1c). LOF genetic variants are indeed expected to impact the viability of MCF10A cells relying on EGFR signaling for growth. Together, these data confirm efficient base editing of endogenous loci in MCF10A cells and our capacity to robustly detect LOF variants in *EGFR*.

### Identification of oncogenic *EGFR* mutations

Toward identifying pathogenic *EGFR* variants that lead to constitutive EGFR signaling in MCF10A cells, we compared sgRNA library distributions in nontreated and EGF-deprived cells. This analysis revealed a spectrum of enriched and depleted *EGFR* variants, largely localized to specific functional protein domains (Fig. 1c and Extended Data Fig. 2b). Among enriched hits, we observed some of the sgRNAs previously identified to impact cell viability, suggesting that cells with decreased viability are outgrown more rapidly by healthy cells in nontreated samples. To focus our analysis on activating mutations in EGFR and avoid biasing our results toward confounding LOF variants impacting MCF10A viability, we chose to only look at variants preserving cell viability in the presence of EGF (log fold change (LFC) > −0.6 when comparing plasmid and late time points) (Fig. 1d and Extended Data Fig. 2b). This approach identified 19 hits that, while spanning the protein, mostly localized to the tyrosine kinase domain, which is responsible for EGFR autophosphorylation and contains the residues most commonly impacted by mutations in NSCLC. Of the 19 hits, 10 were found in ClinVar and labeled as either pathogenic (2) or VUS (8). A further 2 were found in the Catalogue of Somatic Mutations in Cancer (COSMIC) and 7 were not listed in either database (Supplementary Table 1).

Our screen identified well-known rare pathogenic variants such as Thr790Met and Pro596Ser, which are both reported as pathogenic in ClinVar. Surprisingly, the majority of other hits introduce mutations that were previously observed in tumor samples but are currently not considered as pathogenic. For example, Ser720Phe is absent from ClinVar but was previously observed in people with NSCLC^44,45^ and is adjacent to Gly719, which is a residue in the kinase domain commonly impacted by mutations^19^. Several additional hits not considered as pathogenic such as Val765Ile [BE3.9max] and Val769Ile;Ser768Asn [BE3.9max] also affect the αC-helix and the αC–β4 loop, respectively, which are key regulatory structures for the activation of this domain. Lastly, Ile715Val;Lys716Gly [ABE8e] impacts the Lys716 ubiquitination site^46^ and Tyr727Cys;Lys728Glu [ABE8] contains a phosphorylation site^47^, suggesting a role of these post-translational modifications in EGFR regulation and stability.

Interestingly, the co-occurrence of certain screen hits provides insights into the role of autoregulation and phosphorylation in EGFR activation. For example, Asp587 and Lys609 are known to form a salt bridge further stabilized by a loop containing Pro596. This interaction is known to contribute to the autoinhibitory conformation of the inactive receptor^48^, suggesting that its disruption may favor EGFR constitutive activation and constitute a mechanism of oncogenic transformation. To validate this result, we delivered the Asp587Asn [BE3.9max] lentiviral vector to MCF10A cells and sequenced the target genomic locus in nontreated and EGF-deprived cells (Extended Data Fig. 2c). Deep sequencing data analysis confirmed that the predicted Asp587Asn substitution was the most prevalent in both treatment arms. Additionally, alleles harboring this variant appeared enriched under EGF deprivation while the wild-type allele was depleted, confirming that Asp587Asn confers a growth advantage to cells in the absence of EGF.

Our screen also identified three enriched sgRNAs predicted to introduce mutations impacting the Asp314 residue in the extracellular domain and to introduce variants currently classified as VUS. Surprisingly, target sequencing of cells transduced with the Asp314Asn [BE3.9max] construct revealed that the predicted amino acid substitution was depleted under EGF deprivation (Extended Data Fig. 2d). By contrast, another edit located outside of the BE3.9max editing window and introducing the previously uncharacterized Glu317Lys substitution was enriched. This result highlights the importance of validating individual base editing screen hits to account for unanticipated editing products.

Our most surprising result was a cluster of hits found in the C-terminal tail. Among them, we found two exon 25 splice donor mutations predicted to lead to the C-terminal truncation of the receptor after exon 25 (*EGFR*ΔEx26–28). Although the autophosphorylation of this domain is essential for signal transduction of wild-type EGFR stimulated with EGF, it appears not to be required for the downstream signaling of mutant EGFR canonically associated with oncogenesis^49^. On the other hand, different truncated EGFR variants have been identified in glioblastoma^50,51^ but not NSCLC and have been reported to be associated with increased receptor activation because of the loss of an autoinhibitory region of the C-terminal tail^52,53^. Another study showed the activating potential of *EGFR*ΔEx26–28 in NIH-3T3 cells, which is thought to be because of the role of the C-terminal tail in receptor internalization and degradation. This indicates that, although activating *EGFR* mutations are tumor specific, the MCF10A cell line is sensitive to pathogenic EGFR variants spanning cancer types, thereby offering new opportunities to interrogate multiple aspects of EGFR and its role in signaling and cancer.

To validate that C-terminal truncations lead to EGFR activation, we delivered one of the exon-25-truncating sgRNAs into MCF10A cells and let selected cells grow in the absence of EGF for 5 days before measuring their viability (Fig. 1e). We observed that infected cells edited to express *EGFR*ΔEx26–28 displayed higher EGF-independent growth compared to uninfected cells. Deep sequencing of the target site also confirmed the enrichment of alleles with mutated splice site donors, confirming the base editing screen result (Extended Data Fig. 2e).

Taken together, these results demonstrate the ability of base editing mutational scanning to identify known and unknown EGFR-activating and likely oncogenic variants. Notably, in spite of the limited mutational spectrum introduced by each base editor, our screens identify key domains, residues and post-translational modifications likely involved in receptor regulation and stability. In sum, our screens expand the number of likely pathogenic variants in both the tyrosine kinase and the extracellular domains.

### Drug-resistant variant discovery in MCF10A cells

**Fig. 2:**
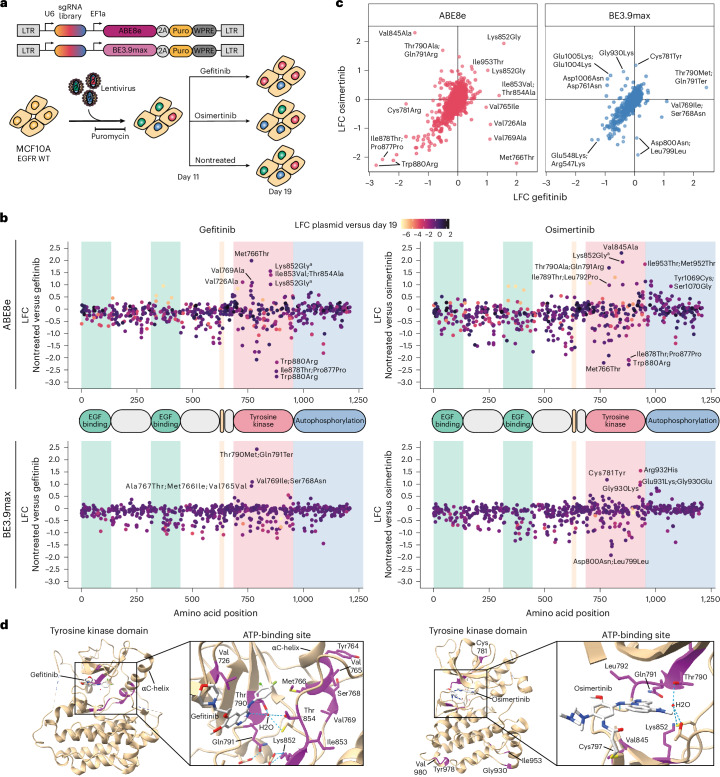
Base editing scanning screens identify EGFR variants resistant to first-generation and third-generation TKIs. **a**, Overview of the base editing constructs and workflow to identify EGFR variants resistant or sensitive to clinically approved TKIs gefitinib or osimertinib. All-in-one base editing libraries packaged in lentivirus were delivered to MCF10A cells in duplicate. Cells were split into three treatment arms on day 11: nontreated, 0.13 μM gefitinib or 0.3 μM osimertinib and harvested on day 19. **b**, Scatter plot showing the LFCs of individual sgRNAs along the EGFR protein between nontreated and drug-treated cells for each base editor and drug treatment. LFCs between the plasmid library and nontreated cells on day 19 are shown as color gradients. Background colors represent EGFR domains. **c**, Scatter plots comparing the LFCs of sgRNAs in cells treated with either gefitinib or osimertinib for each base editor. Nontargeting and essential splice site controls are not shown, neither are sgRNAs with LFCs < −1.5 between plasmid and day 19. **d**, Three-dimensional structures of the EGFR tyrosine kinase domain in complex with gefitinib (left; Protein Data Bank (PDB) 2ITY) or osimertinib (right; PDB 6JXT) as visualized in ChimeraX. Hits of the respective MCF10A screens are shown in magenta and TKI molecules are shown in gray. ^a^Validation of the Lys852Gly [ABE8e] sgRNA showed that the main variant conferring drug resistance was Lys852Glu (Extended Data Fig. 5d). Source data

Encouraged by these results, we then set out to expand our screening approach to evaluate the sensitivity of EGFR variants to clinically approved TKIs in the MCF10A cell line. We repeated both base editing screens in the same conditions but replaced the EGF depletion step with either gefitinib or osimertinib treatments, which are first-generation and third-generation TKIs, respectively (Fig. 2a). Both drugs compete with adenosine triphosphate (ATP) to bind the tyrosine kinase active site but through different inhibition mechanisms^10,13^, which also raises the possibility to dissect drug-specific resistance mechanisms. We deployed our base editing variant-scanning methodology to explore this.

**Extended Data Fig. 3:**
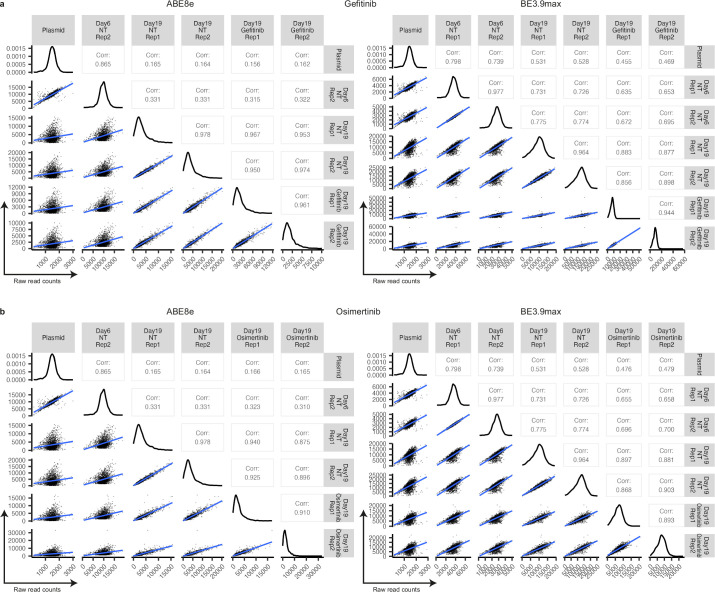
sgRNA library distributions and sample correlations for the drug resistance screens in MCF10A cells. (**a**,**b**) Raw read counts and Pearson correlation coefficients are shown for all samples in the gefitinib (**a**) and osimertinib (**b**) resistance screens.

We delivered the BE3.9max and ABE8e *EGFR* variant-scanning libraries to MCF10A cells at low MOI and selected for infected cells with puromycin. We then applied osimertinib or gefitinib treatment for 8 days before sgRNA library preparation and sequencing. Library quantification was performed as previously and we confirmed high replicate correlation (Extended Data Fig. 3a,b).

**Extended Data Fig. 4:**
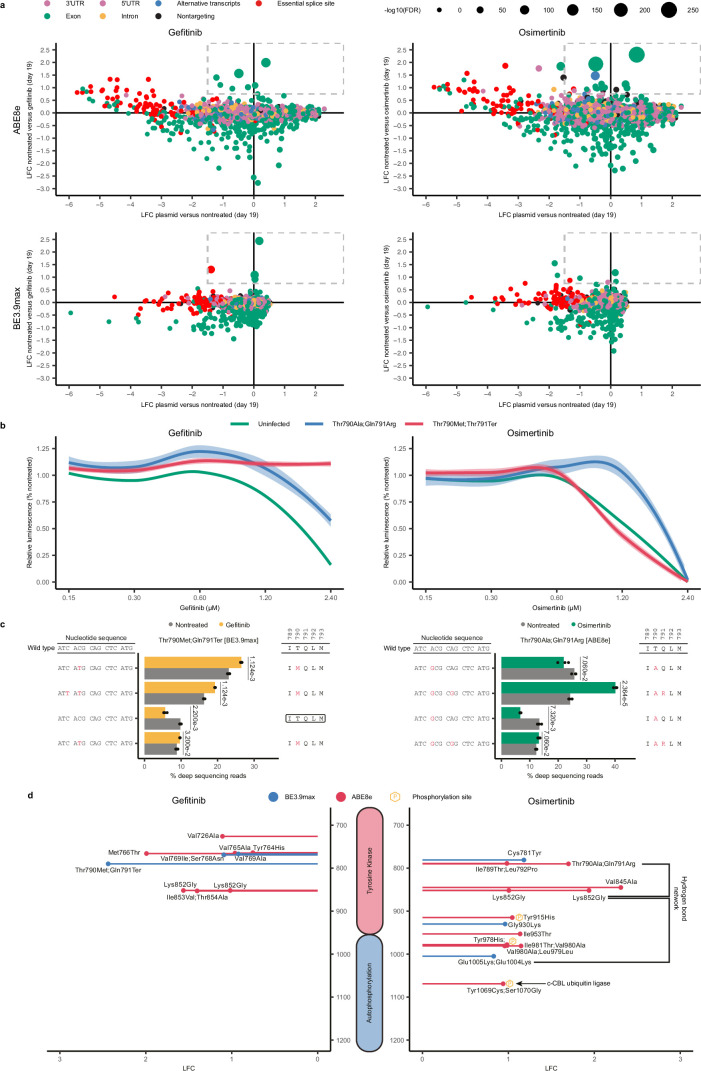
Impact of individual sgRNAs on cell viability and drug response in the base editing drug resistance screens in MCF10A cells. (**a**) Scatter plot comparing the individual sgRNA LFCs between plasmid and day 19 and between nontreated and TKI-treated samples. Negative-log FDR values for drug resistance sgRNAs are shown. Dashed squares represent the hit inclusion criteria: LFC (plasmid vs day 19) > −1.5 and LFC (nontreated vs TKI-treated) > 0.75. (**b**) TKI response validation of drug resistance screen hits in MCF10A cells. Relative viabilities of edited and unedited MCF10A cells after gefitinib or osimertinib treatment as measured by the CellTiter-Glo assay. Cells were infected in triplicates and treated for 5 days with 0, 0.15, 0.3, 0.6, 1.2 or 2.4 μM of the respective drug before viability measurements. Solid lines represent replicate means and shaded areas represent the standard error. (**c**) Deep sequencing data for individual sgRNA validation in MCF10A cells. Cells were infected with the indicated sgRNA and base editor pairs in three biological replicates (*n* = 3) and later split into control and treatment arms with gefitinib or osimertinib. The percentages of reads corresponding to the four most represented alleles at each target site are shown for nontreated and drug-treated cells. Nucleotide sequences represent the coding DNA strand and black squares denote wild-type *EGFR* sequences. Holm-adjusted *P* values are shown for two-sided *t*-tests. (**d**) Diagram showing combined ABE8e and BE3.9max screen hits along the EGFR tyrosine kinase and C-terminal domains for cells treated with gefitinib or osimertinib. Hits are defined as sgRNAs with LFC > −1.5 between plasmid and day 19 and LFC > 0.75 between nontreated and TKI-treated cells. Notable post-translational modifications and intramolecular interactions are shown. (*) Validation of the Lys852Gly [ABE8e] sgRNA showed that the main variant conferring drug resistance was Lys852Glu (Extended Data Fig. 5d).

We started our analysis by comparing sgRNA enrichment between treated and nontreated samples while accounting for variant fitness (Fig. 2b and Extended Data Fig. 4a). Encouragingly, we observed that, with both drug treatments, a majority of enriched hits were located in the tyrosine kinase domain and included well-known drug-resistant variants. For example, Thr790Met is known as the most prevalent gefitinib-resistant variant and we observed a strong enrichment of the Thr790Met;Gln791Ter [BE3.9max] sgRNA under gefitinib treatment. Importantly, this same variant is not enriched under osimertinib treatment, which is expected as this molecule was specifically developed to counter its emergence. Unexpectedly, osimertinib selection led to the enrichment of different predicted variants at the same position, namely Thr790Ala;Gln791Arg [ABE8e].

We set out to validate this result in a follow-up experiment and measured the viability of MCF10A cells infected with individual sgRNA and base editor pairs followed by TKI treatment for 5 days (Extended Data Fig. 4b). As predicted by the screen, cells infected with Thr790Ala;Gln791Arg [ABE8e] showed higher resistance to osimertinib than to gefitinib, although neither variants are listed in ClinVar, while Thr790Met;Gln791Ter [BE3.9max] showed the opposite resistance profile. Deep sequencing of the genomic target site after drug treatment revealed the enrichment of the Thr790Met edit in the case of gefitinib and the Thr790Ala;Gln791Arg double-mutant allele in the case of osimertinib. In this case, the alleles containing Thr790Ala alone were depleted, suggesting that Gln791Arg is likely driving the resistance phenotype (Extended Data Fig. 4c). This confirms both the specificity and the sensitivity of our base editing variant screening approach and highlights the need to understand drug resistance at single-base resolution.

**Extended Data Fig. 5:**
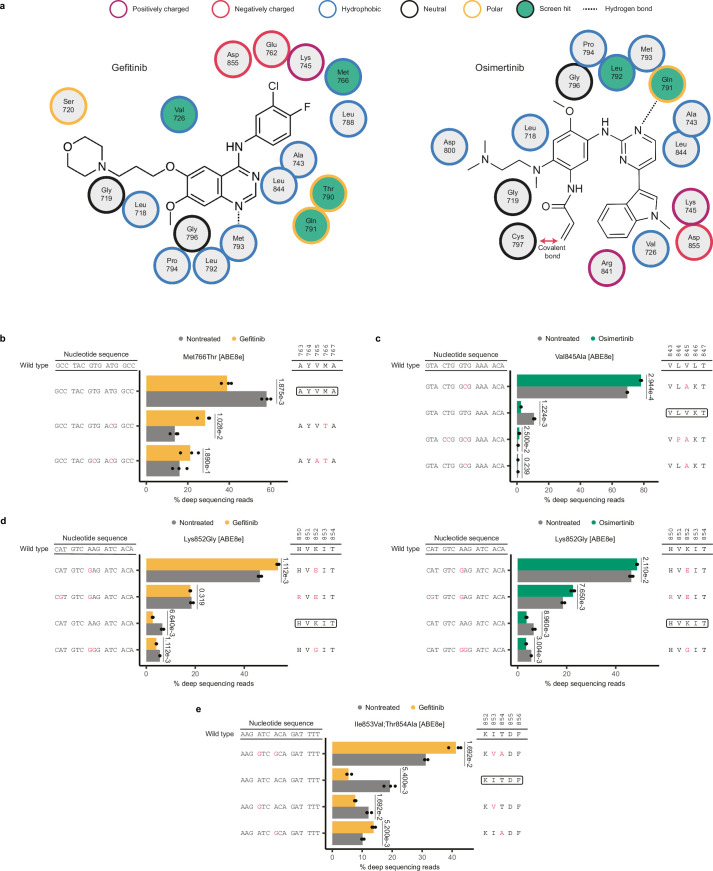
Structural relevance and validation of the MCF10A drug resistance screens hits. (**a**) Two dimensional projections of the EGFR tyrosine kinase domain residues in contact with gefitinib (PDB #2ITY) and osimertinib (PDB #6JXT). All residues within 4 Å around the ligands are shown with hits of the respective MCF10A screens colored in green. Circle colors represent amino acid physical properties. (**b**–**e**) Deep sequencing data for individual sgRNA validation in MCF10A cells. Cells were infected with the indicated sgRNA and base editor pairs in three biological replicates (*n* = 3) and later split into control and treatment arms with gefitinib or osimertinib. The percentages of reads corresponding to the four most represented alleles at each target site are shown for nontreated and drug-treated cells. Nucleotide sequences represent the coding DNA strand and black squares denote wild-type *EGFR* sequences. Holm-adjusted *P* values are shown for two-sided *t*-tests.

Under gefitinib selection, all enriched screen hits were located in the tyrosine kinase domain and more specifically around the ATP-binding pocket, which constitutes the binding site of both drugs (Fig. 2b, Extended Data Fig. 4d and Supplementary Table 2). In addition to the Thr790 gatekeeper residue, we identified previously unknown resistant mutations found to affect Val726, Met766 and Thr854, which are all in direct contact with the receptor-bound molecule (Fig. 2d and Extended Data Fig. 5a). We speculate that mutations impacting these residues can directly affect the binding affinity of gefitinib. Importantly, Thr854Ala is classified as VUS, Val726Ala is not listed in either database and Met766Thr has been shown to be resistant to gefitinib in vitro^54^ but is not listed in ClinVar. To validate this hit, we delivered a lentiviral construct containing the Met766Thr [ABE8e] sgRNA to MCF10A cells and treated them with gefitinib for 7 days, under similar conditions to the screening protocol. Deep sequencing of the target exon revealed an enrichment of reads containing the Met766Thr edit under gefitinib treatment while wild-type alleles were depleted, thus confirming the resistance phenotype (Extended Data Fig. 5b).

Similarly to gefitinib, the top three hits for osimertinib affect residues found in the ATP-binding pocket of the tyrosine kinase domain (Fig. 2d, Extended Data Fig. 5a and Supplementary Table 3). For example, Val845Ala [ABE8e] interacts with the Phe795 and Gly796 residues adjacent to Cys797, which is the osimertinib covalent binding site, hinting at a resistance mechanism involving this residue. Although this mutant is not listed in ClinVar, the similar Val845Leu variant has conflicting reports of pathogenicity in the database. We set out to confirm this phenotype by individual sgRNA delivery and target exon sequencing after drug treatment. This revealed the enrichment of the Val845Ala edit and a strong depletion of wild-type alleles under osimertinib selection, thus validating this hit (Extended Data Fig. 5c).

The remaining hits were found throughout the tyrosine kinase and C-terminal domains, highlighting distinct EGFR regulation mechanisms. For example, enriched sgRNAs were found to affect Lys852 and Gln791, which interact with each other in the osimertinib-bound receptor to form a hydrogen-bond network with residues Asp1012 and Asp1014 of the C-terminal domain. Neither of these variants are listed in ClinVar, although perturbing this network by replacing Gln791 with a hydrophobic residue has been predicted to destabilize osimertinib binding^55^. Interestingly, in the inactive conformation, Lys852 is also known to directly interact with another hit, Glu1005, which is part of a C-terminal ‘electrostatic hook’ that inhibits the kinase domain activity. Mutations impacting Glu1005 and Asp1006 have been shown to increase the activity of unstimulated EGFR in vitro^56^, thus potentially promoting the observed resistance phenotype. Targeted amplicon sequencing-based validation of the Lys852Gly [ABE8e] hit confirmed the resistance phenotype with both drugs (Extended Data Fig. 5d). However, contrary to our base editing outcome prediction, deep sequencing data revealed the enrichment of alleles substituting the lysine at position 852 with a glutamine instead of the expected glycine.

Taken together, the base editing variant-scanning results highlight key intramolecular interactions between EGFR residues involved in enzymatic activity regulation, resulting in new and intricate insights into drug-dependent resistance mechanisms. Interestingly, while top resistant mutations for both drugs are found to impact residues in the ATP-binding pocket, osimertinib-resistant hits are also found in the C-terminal domain, hinting at both shared and divergent resistance mechanisms between the drugs. These insights, substantiated by clinical validation, may in the future help clinical decision making.

### Variant scanning allows for drug prioritization

In addition to drug-resistant variants, our screening data identify variants that likely increase drug sensitivity. Such candidates are revealed by sgRNAs that are depleted under drug selection. Indeed, when comparing the LFCs of each sgRNA and base editor pair under gefitinib and osimertinib selection, we observe that variant sensitivities vary between both drugs with some mutations leading to opposite effects (Fig. 2c). For example, we observed that Val845Ala [ABE8e] appeared strongly resistant to osimertinib but sensitive to gefitinib. On the other hand, other variants such as Val726Ala [ABE8e], Val769Ala [ABE8e] and Met766Thr [ABE8e] appeared to confer different levels of resistance to gefitinib but to be sensitive to osimertinib. All of these variants are located within the ATP-binding pocket, which suggests that their differential drug sensitivities resulted from specific direct interactions with each molecule.

We also identified variants that appeared to be resistant to both drugs, such as Lys852Glu [ABE8e], which is currently not listed in ClinVar or COSMIC. This residue is known to be involved in reciprocal interactions with residues of the C-terminal tail and to contribute to EGFR autoinhibition. This suggests that common resistance mechanisms can emerge when EGFR autoinhibition is disrupted. Taken together, these results provide new insights into EGFR variant-dependent drug sensitivities, which may help guide therapeutic decisions in the future for clinicians faced with EGFR variants for which clinical data are currently absent.

### Distinct sensitivities of primary and compound *EGFR* mutations

In cancer patients, TKIs are used to counteract the activity of hyperactive EGFR mutants, such as the common Leu858Arg substitution or exon 19 deletion^20^. We set out to apply our base editing variant-scanning pipeline to evaluate the impact of secondary EGFR mutations on drug sensitivity in the NSCLC-derived PC-9 cell line. These cells represent an attractive model because they are sensitive to TKIs and harbor *EGFR*ΔGlu746–Ala750, which is the most prevalent EGFR deletion in lung cancer^45^. Additionally, the introduction of the Thr790Met variant in PC-9 cells with base editing has previously been shown to lead to a strong gefitinib resistance phenotype^57^. We, thus, performed a base editing scanning screen using the same *EGFR*-targeting sgRNA library and experimental and computational workflows as demonstrated for MCF10A cells.

**Extended Data Fig. 6:**
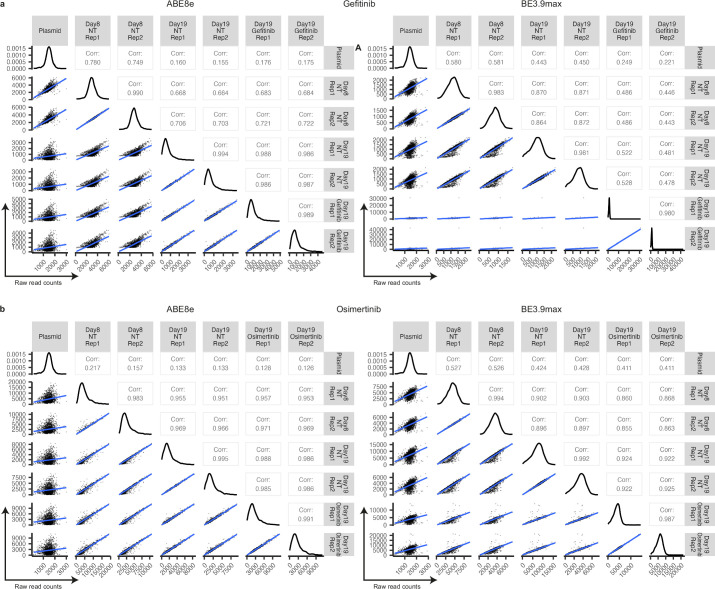
sgRNA library distributions and sample correlations for the drug resistance screens in PC-9 cells. (**a**,**b**) Raw read counts and Pearson correlation coefficients are shown for all samples in the gefitinib (**a**) and osimertinib (**b**) resistance screens.

**Extended Data Fig. 7:**
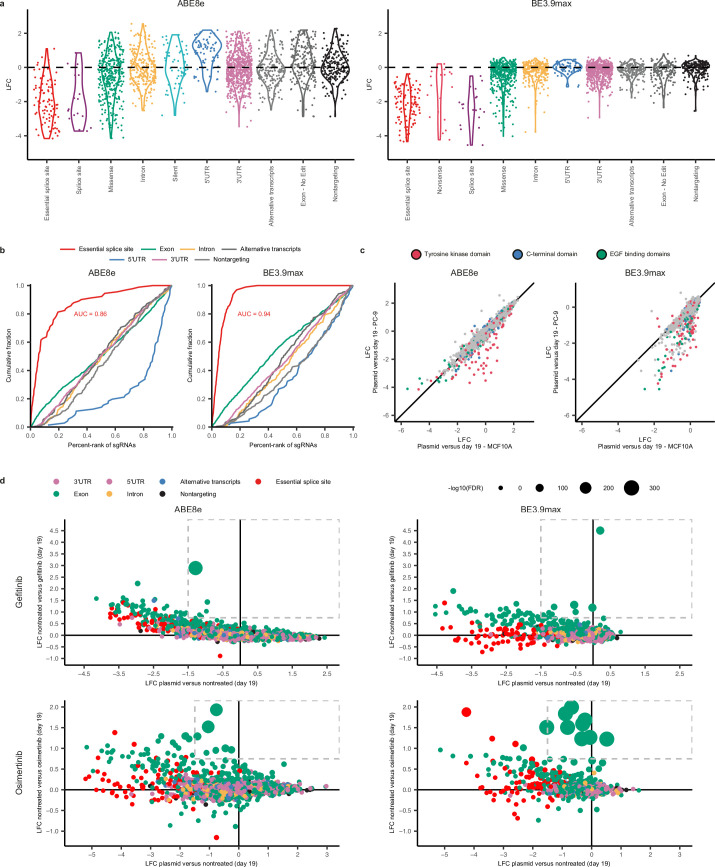
Impact of individual sgRNAs on cell viability and drug response in the base editing screens in PC-9 cells. (**a**) LFCs of individual sgRNAs between plasmid and day 19 (nontreated) for each target category or predicted mutation type. (**b**) ROC curves for each target category or predicted mutation type between plasmid and day 19 (nontreated). AUC for essential splice site sgRNAs are shown in red. (**c**) Comparisons of the LFCs between plasmid and day 19 library distributions in MCF10A and PC-9 cells. Only *EGFR*-targeting sgRNAs are represented, guides targeting the tyrosine kinase or C-terminal domains are highlighted in red and blue, respectively. (**d**) Scatter plot comparing the individual sgRNA LFC between plasmid and day 19 and between nontreated and TKI-treated samples. Negative-log FDR values for drug resistance sgRNAs are shown. Dashed squares represent the hit inclusion criteria: LFC (plasmid vs day 19) > −1.5 and LFC (nontreated vs TKI-treated) > 0.75.

We started our analysis by comparing sgRNA counts between plasmid and day 19 conditions and confirming high replicate correlation, no shift in the negative control sgRNA population and the depletion of positive control sgRNAs targeting essential splice sites (Extended Data Figs. 6a,b and 7a,b). As was the case in the wild-type *EGFR* MCF10A cells, in *EGFR*-mutant PC-9 cells, we also identified a spectrum of *EGFR* secondary mutations imparting fitness effects. While many of the fitness-altering mutations were shared between MCF10A and PC-9 cells, we identified unique subsets for each cell line (Extended Data Fig. 7c). Interestingly, tyrosine kinase variants appear to have a stronger impact on viability in *EGFR*-mutant PC-9 compared to wild-type *EGFR* MCF10A cells, which we speculate could be because of reduced resilience of mutant EGFR or oncogene addiction.

**Fig. 3:**
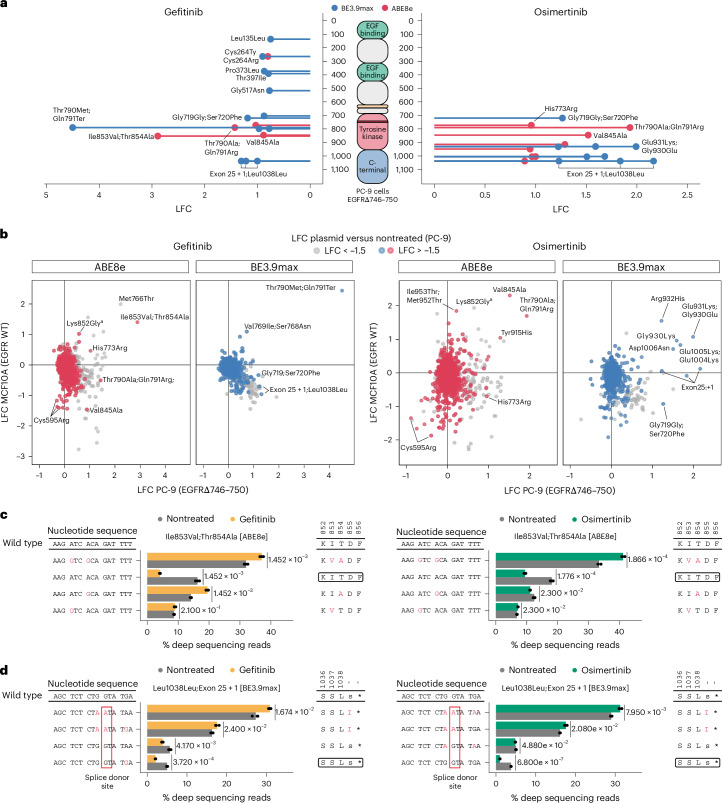
Base editing scanning screens identify unique and shared drug-resistant variants between cell lines harboring WT *EGFR* and *EGFR*ΔGlu746–Ala750. **a**, Diagram showing combined ABE8e and BE3.9max screen hits along the EGFR protein in PC-9 cells. Hits are defined as sgRNAs with LFCs > −1.5 between plasmid and day 19 and LFC > 0.75 between nontreated and EGF-deprived cells. **b**, Scatter plots comparing the LFCs of sgRNAs in MCF10A (WT *EGFR*) and PC-9 (*EGFR*ΔGlu746–Ala750) cell lines treated with gefitinib or osimertinib. sgRNAs with LFC < −1.5 between plasmid and day 19 in PC-9 cells are shown in gray. Nontargeting and essential splice site controls are not shown. **c**,**d**, *EGFR-*targeted amplicon sequencing data for individual sgRNA validation in PC-9 cells. PC-9 cells were infected with sgRNAs Ile853Val;Thr854Ala [ABE8e] (**c**) or Leu1038Leu;Exon 25 + 1 [BE3.9max] (**d**) in three biological replicates and later split into control and treatment arms with gefitinib or osimertinib. The percentages of reads corresponding to the four most represented alleles at each target site are shown for nontreated and drug-treated cells. Nucleotide sequences represent the coding DNA strand and amino acid sequences correspond to the annotated EGFR residues. Black and red squares denote WT *EGFR* and splice donor sites, respectively. Holm-adjusted *P* values are shown for two-sided *t*-tests. Asterisks within barplots in **d** refer to the stop codon. ^a^Validation of the Lys852Gly [ABE8e] sgRNA showed that the main variant conferring drug resistance was Lys852Glu (Extended Data Fig. 8d). Source data

**Extended Data Fig. 8:**
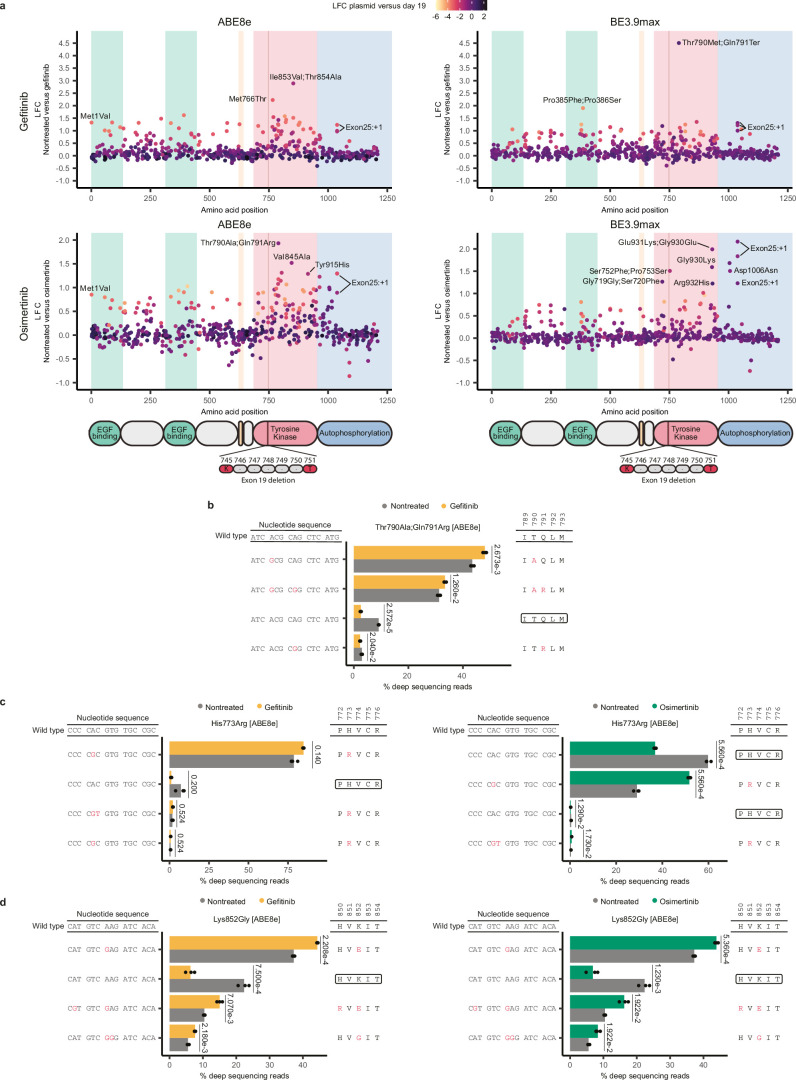
Base editing mutational scanning screens identify EGFR variants resistant to gefitinib and osimertinib in PC-9 cells. (**a**) Scatter plot showing the LFCs of individual sgRNAs along the EGFR protein between nontreated and drug-treated cells for each base editor and drug treatment. LFCs between the plasmid library and nontreated cells on day 19 are shown in color gradients. Background colors represent EGFR domains. (**b**–**d**) Deep sequencing data for individual sgRNA validation in PC-9 cells. Cells were infected with the indicated sgRNA and base editor pair in three biological replicates (*n* = 3) and later split into control and treatment arms with gefitinib or osimertinib. The percentages of reads corresponding to the four most represented alleles at each target site are shown for nontreated and drug-treated cells. Nucleotide sequences represent the coding DNA strand and black squares denote wild-type *EGFR* sequences. Holm-adjusted *P* values are shown for two-sided *t*-tests.

Next, we set out to characterize the impact of EGFR secondary mutations on TKI drug resistance (Fig. 3a and Extended Data Figs. 7d and 8a). Similarly to MCF10A cells, we observed that the most enriched gefitinib-resistant hits are located in the tyrosine kinase domain, while top osimertinib hits span both the tyrosine kinase and the C-terminal domains. We, thus, continued our analysis by comparing shared hits between both cell lines. While this revealed a broad range of shared and distinct variants spanning *EGFR*, we noticed that the most commonly impacted positions appeared to be the same regardless of the initial *EGFR* genotype (Fig. 3b). For example, in both cell lines, Thr790Met;Gln791Ter [BE3.9max] and Ile853Val;Thr854Ala [ABE8e] are strongly enriched under gefitinib treatment while Thr790Ala;Gln791Arg [ABE8e] and Val845Ala [ABE8e] are enriched with osimertinib.

To confirm a selection of these insights, we delivered the Ile853Val;Thr854Ala [ABE8e] to PC-9 cells and treated infected cells with gefitinib or osimertinib for 7 days. Deep sequencing of the targeted region revealed that reads corresponding to the unedited alleles were strongly depleted under drug treatment, thus confirming efficient growth inhibition in unedited cells, as also previously observed in MCF10A cells (Fig. 3c and Extended Data Fig. 5e). By contrast, we observed with both drugs the enrichment of reads containing the Ile853Val;Thr854Ala double edit while Thr854Ala alone was enriched under gefitinib selection but not osimertinib. Thr854Ala is currently classified as VUS and has been previously observed in people treated with first-generation TKIs^58^. Our data, thus, suggest that these residues are important for the activity of both drugs, whereas Thr854Ala is sufficient to drive resistance to gefitinib but not osimertinib.

We then compared sgRNAs that were differentially enriched across the two cell lines. For example, Thr790Ala;Gln791Arg [ABE8e] and Gly719Gly;Ser720Phe [BE3.9max] appeared to only confer gefitinib resistance in PC-9 cells but not in MCF10A, suggesting the existence of different tyrosine kinase conformations and ligand interactions between both cell lines. In a follow-up experiment, we delivered the Thr790Ala;Gln791Arg [ABE8e] sgRNA to PC-9 cells and confirmed the enrichment of the Thr790Ala variant under drug treatment and its role in gefitinib resistance in this cell line (Extended Data Fig. 8b). Similarly, His773Arg [ABE8e] is currently classified as likely pathogenic and was enriched with both drugs in PC-9 cells but not in MCF10A. Validation of this construct confirmed the resistant phenotype under osimertinib selection but the allele enrichment was not statistically significant after gefitinib treatment, possibly because of a lower resistance to this molecule (Extended Data Fig. 8c).

Surprisingly, with both drugs and both base editors, we noticed the enrichment of sgRNAs predicted to impact the exon 25 splice donor and leading to the *EGFR*ΔEx26–28 truncation. Interestingly, this truncation was found to drive EGFR activation in our MCF10A screen. However, it appears to confer drug resistance only in PC-9 cells, hinting at a resistance mechanism requiring previous receptor hyperactivation or a specific receptor conformation resulting from the ΔGlu746–Ala750 deletion. Individual sgRNA follow-up experiments confirmed an enrichment of edited alleles with a mutated splice donor site under gefitinib and osimertinib treatment (Fig. 3d). Taken together, these data confirm that the secondary truncation of a constitutively active EGFR variant such as *EGFR*ΔGlu746–Ala750 is able to maintain its downstream signaling and might constitute a possible mechanism of resistance to first-generation and third-generation TKIs.

Together, these results highlight the importance of evaluating drug resistance variants in relevant genomic contexts, including pre-existing *EGFR* mutations, and confirm the relevance of sequencing the target site to validate base editor screen hits.

### Interrogation of patient-derived variants with prime editing

**Extended Data Fig. 9:**
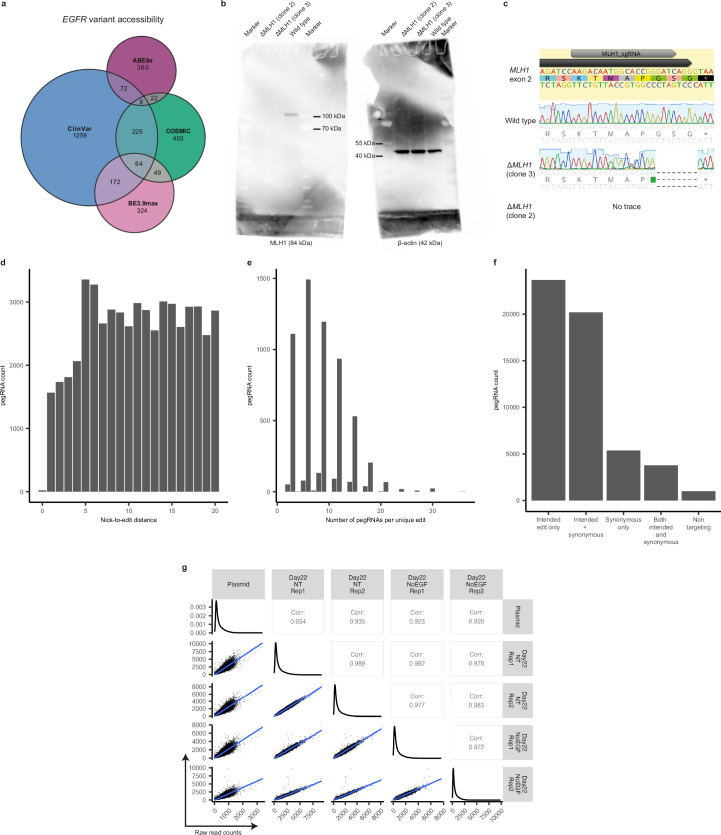
*MLH1* knock-out cell line validation and EGFR-targeting epegRNA library design. (**a**) Venn diagram showing the numbers of *EGFR* coding variants listed in the ClinVar and COSMIC databases that can be introduced by the ABE8e and BE3.9max base editors. All possible sgRNAs with an NGG PAM targeting *EGFR* exons were considered and editing outcomes were predicted using the base editor design tool from Hanna et al.^33^. (**b**,**c**) (**b**) Western Blot analysis of MLH1 protein expression and (**c**) Sanger sequencing of the *MLH1* endogenous gene locus in two clonal cell lines derived from the transfection of MCF10A cells with a plasmid expressing the Cas9 enzyme and a sgRNA targeting *MLH1* exon 2. Clone 3 was used for all subsequent experiments. The data are representative of a single experiment. (**d**) Bar plot representing the distance between the nCas9-induced nick and intended edit across the pegRNA library. A distance of zero corresponds to deletions overlapping the nick-adjacent nucleotide. (**e**) Histogram representing the number of pegRNAs attributed to each unique edit in the library. The pegRNAs sharing the same intended mutations with or without additional synonymous mutation are considered separately. (**f**) Bar plot representing the number of pegRNAs in the library according to the type of introduced mutation. Intended edits are, when possible, associated with an additional synonymous mutation (intended + synonymous). For each of these, control pegRNAs introducing only the intended edit (intended edit only) or the synonymous mutation (synonymous only) are also added to the library. Some edits can correspond to both intended and synonymous mutations (both intended and synonymous). (**g**) Library distributions and sample correlations for the prime editing EGFR activation screen in MCF10A cells. Raw barcode counts and Person correlation coefficients are shown for all samples.

Base editing screens enable the sensitive identification of phenotype-inducing variants at accessible nucleotides but remain limited in terms of the mutation spectrum they can introduce. In particular, ABE8e and BE3.9max coupled with wild-type SpCas9 can introduce only about 17.6% and 18.6% of coding *EGFR* variants listed in ClinVar and COSMIC, respectively (Extended Data Fig. 9a). While this limited capacity to induce desirable edits is in part because of protospacer-adjacent motif (PAM) restriction that can be circumvented through alternative Cas enzymes^59^, most of the mutations simply cannot be introduced because of the impossibility of base editing chemistry to convert between all codons. For instance, one of the most prevalent EGFR-activating variants is Leu858Arg, which is not found in our base editing screens because its codon can only be targeted by BE3.9max, where it is predicted to introduce a synonymous mutation.

**Fig. 4:**
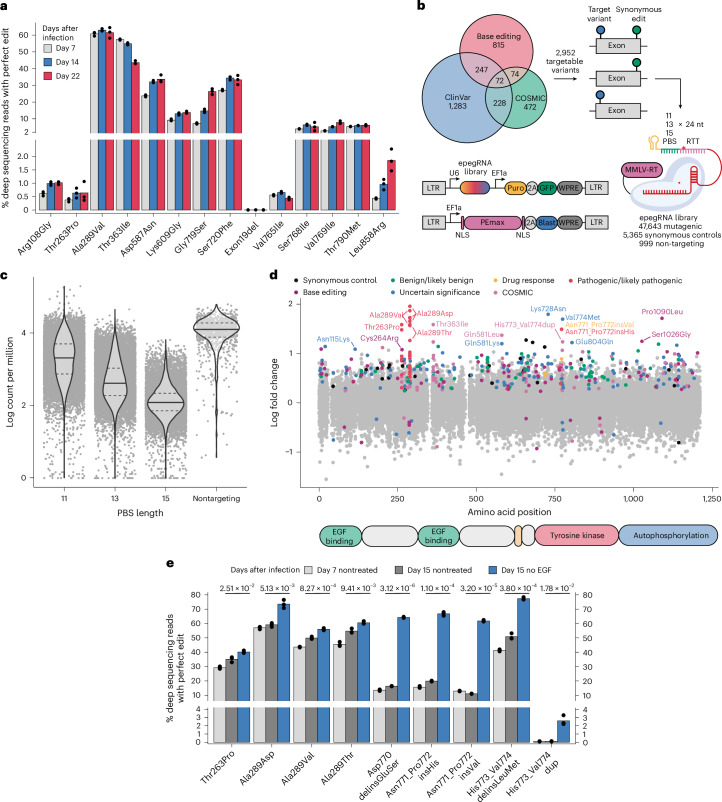
Prime editing scanning screen of patient-derived variants identify EGFR-activating mutations in MCF10A cells. **a**, Prime editing efficiency in MCF10A^∆MLH1^ cells expressing the PEmax enzyme. *EGFR*-targeting pegRNAs introducing 14 pathogenic variants were designed using PRIDICT2.0 (ref. ^68^). Cells were infected with the indicated pegRNAs in three biological replicates and editing efficiencies were measured by deep sequencing of the respective target sites on days 7, 14 and 22 after transduction. **b**, Schematic of the prime editing screening approach. Variants impacting *EGFR* exons or UTRs and shorter than 11 nucleotides were obtained from ClinVar, COSMIC and the base editing screens. Whenever possible, additional synonymous mutations were added to the adjacent codon and/or the Cas9 PAM sequence was mutated to increase prime editing efficiency. For each combination, pegRNAs introducing only the intended edit or the synonymous mutation were added to the library as controls. Each pegRNA was designed with three different PBS lengths and a 24-nt RTT. Lentiviral pegRNA library and PEmax delivery vectors are shown. **c**, Violin plot showing individual barcode counts per million derived from the deep sequencing of the final pegRNA library (*n* = 1) according to the PBS length of the corresponding pegRNAs. Solid horizontal lines represent population medians and dashed lines represent 0.25 and 0.75 quantiles. Generalized linear model (Poisson regression) coefficients: PBS length, −3.26 × 10^−1^ (*P* < 2 × 10^−16^); intercept = 9.82 (*P* < 2 × 10^−16^) on 53,007 degrees of freedom. **d**, LFCs of individual pegRNAs between nontreated and EGF-deprived cells along the EGFR protein. Colors denote the variant classifications for pegRNAs with −log_10_(FDR) > 2. **e**, Deep sequencing data for individual pegRNA validation. PEmax-expressing MCF10A^∆MLH1^ cells were infected with lentivirus encoding the indicated pegRNAs in triplicate and deprived of EGF from day 7 to day 15. The percentages of reads that correspond to perfect edits, including additional synonymous mutations, are shown, as well as *P* values for two-sided *t*-tests between nontreated and EGF-deprived samples on day 15. Source data

To explore a more clinically relevant mutational space, we set out to leverage prime editing, which can introduce all possible base substitutions, as well as short insertions and deletions^35^. First, we established an MCF10A cell line harboring an *MLH1* gene knockout, which has been shown to drastically improve prime editing efficiency^36^, and expressing the PEmax enzyme (Extended Data Fig. 9b,c). We then tested prime editing efficiency in this cell line using lentiviral delivery of engineered pegRNAs (epegRNAs, henceforth referred to as pegRNAs)^37^ designed to introduce 14 *EGFR* mutations found in variant databases or our base editing screen results. The quantification of edited allele fractions by deep sequencing on days 7, 14 and 22 after transduction revealed drastically different prime editing efficiencies across targets, with 5 pegRNAs yielding less than 2% of edited alleles while 5 others performed beyond 25% at day 22 (Fig. 4a). The highest editing efficiency was 66%, obtained for the Ala289Val variant. Interestingly, most targets appeared to have reached editing saturation at day 14 with the exception of Gly719Ser, which progressed from 7.2% to 26.3% between days 7 and 22, potentially suggesting a growth advantage of cells with this variant in presence of EGF. By contrast, the edited fraction of Thr363Ile, a mutation previously reported in glioblastoma, decreased over time, suggesting a negative impact on cell fitness.

Encouraged by these results, we designed a pegRNA library introducing all variants impacting *EGFR* exons and untranslated regions (UTRs) listed in the ClinVar and COSMIC databases (Fig. 4b). With the goal of directly comparing base editing and prime editing technologies, we additionally included 815 variants introduced in our previous base editing screens. Of the 3,191 unique *EGFR* variants found, 2,952 (~92%) could be introduced by prime editing with a maximum distance of 20 nucleotides between the edit and nick site. The remaining 239 variants could not be targeted because no spacer was available in close proximity or because the corresponding pegRNAs contained poly(T) or BsmBI restriction sites that would preclude their expression or cloning, respectively. For each accessible variant, barcoded pegRNAs were designed with different combinations of primer binding site (PBS) lengths to understand how this parameter impacts library production and maximize our chances of successful editing. Whenever possible, additional synonymous mutations were added alongside the desired edit, which has been shown to increase prime editing efficiency^60^. In these cases, pegRNAs harboring only the intended edit or the synonymous variant were also added to the library to control for unexpected effects on these combinations. The resulting library was cloned into a lentiviral vector expressing a puromycin resistance marker and green fluorescent protein (GFP) (Fig. 4b and Extended Data Figs. 9d–f).

Upon sequencing the pegRNA plasmid library, we observed a bias between *EGFR*-targeting pegRNAs with different PBS lengths (Fig. 4c). Interestingly, this effect is not observed in the case of nontargeting pegRNAs that have a scrambled extension not complementary to their spacer. We speculate that a longer PBS negatively impacts pegRNA cloning because the PBS, unlike the reverse transcription template (RTT), is complementary with the pegRNA spacer, which could result in the formation of secondary structures during oligo library amplification or cloning. While this bias should be considered when designing large pegRNA libraries, especially when including a broad range of PBS lengths, we nonetheless decided to move forward with our small library, as its global bias was minimal (skew ratio = 7.7).

**Extended Data Fig. 10:**
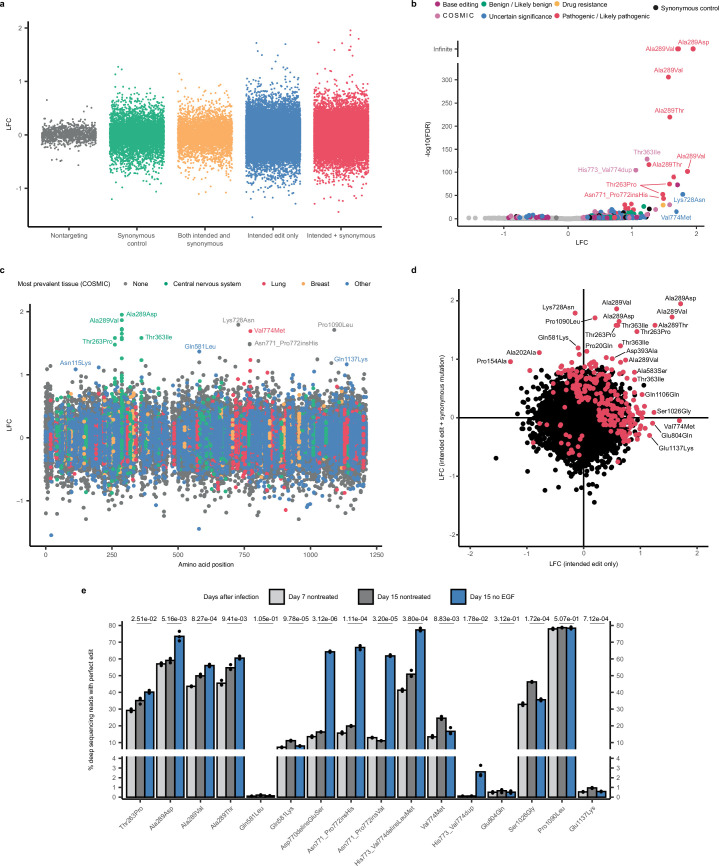
Prime editing scanning screen for EGFR activation identifies enriched variants conferring EGF-independent growth. (**a**) Scatter plot representing the LFCs of individual pegRNAs between the nontreated and EGF-deprived arms of the screen for each mutation category. Intended edits can be introduced along with an additional synonymous mutation (intended + synonymous). For each of these, additional control pegRNAs introducing only the intended edit (intended edit only) or the synonymous mutation (synonymous only) are also added to the library. Some edits can correspond to both intended and synonymous mutations (both intended and synonymous). (**b**) Volcano plot showing the LFCs and negative-log FDR of individual pegRNAs between nontreated and EGF-deprived cells. Colors represent the clinical significance of the corresponding variants. (**c**) Scatter plot showing the LFCs of individual pegRNAs between nontreated and EGF-deprived cells along the EGFR protein. Colors denote the most prevalent tissues for each variant as reported in the COSMIC database. (**d**) Scatter plot comparing the LFCs of pegRNAs introducing the same intended edit with or without additional synonymous mutation. Only pegRNAs with matching spacers and primer binding site (PBS) lengths are shown. The pegRNAs with a -log10(FDR) > 2 in either category are shown in red. (**e**) Deep sequencing data for individual pegRNA validation. PEmax-expressing MCF10A^∆*MLH1*^ cells were infected with the indicated pegRNAs in three biological replicates (*n* = 3) and later split into control and treatment arms deprived of EGF. The percentages of reads correspond to perfect edits, including additional synonymous mutations. The *P* values are shown for two-sided *t*-tests between nontreated and EGF-deprived samples.

We then set out to introduce our library of patient-derived mutations in EGF-dependent MCF10A cells and assess their EGFR activation potential. We delivered our pegRNA library to MCF10A^∆MLH1^ cells stably expressing PEmax^36^ and let them accumulate edits for 14 days before EGF deprivation. Cells were harvested after 8 days of selection followed by pegRNA barcode sequencing. After confirming high replicate correlations (Extended Data Fig. 9g), we compared pegRNA counts between the nontreated and EGF-deprived arms of the screen. As expected, we observed no change in the distributions of nontargeting constructs, while the most enriched pegRNAs introduced intended edits with or without additional synonymous mutations (Extended Data Fig. 10a).

Our screen revealed many known and surprising unknown hits. For example, we observed an enrichment of pegRNAs introducing multiple pathogenic mutations affecting the Ala289 and Thr263 residues in the extracellular domain (Fig. 4d and Extended Data Fig. 10b). Additionally, we identified Thr363Ile, a variant present in COSMIC and predicted to be pathogenic but absent from ClinVar. In these cases, individual pegRNAs of different extension lengths introducing the same edit were enriched together, suggesting that these did not arise through technical noise. These extracellular domain mutations are not frequently found in epithelial cell-derived breast cancers^61^ (the origin of MCF10A cells) but are most frequently found in glioblastoma, with Ala289Val and Ala289Asp being the most common^62,63^ (Extended Data Fig. 10c).

Surprisingly, our screen did not capture common mutations impacting the tyrosine kinase domain such as Leu858Arg or exon 19 deletions, likely because of low editing efficiencies observed for these variants. By contrast, our screen identified individual pegRNAs introducing exon 20 insertions affecting the αC–β4 loop of the tyrosine kinase domain. These constitute a largely uncharacterized category of oncogenic mutations found in lung cancer and associated with resistance to first-generation and third-generation TKIs^20,64^. While Asn771_Pro772insVal and Asn771_Pro772insHis are classified as drug resistant and pathogenic, respectively, we also identify variants not listed in ClinVar such as His773_Val774 duplication or His773_Val774delinsLeuMet.

To validate a selection of our hits, we delivered individual pegRNAs to PEmax-expressing MCF10A^∆MLH1^ cells and measured the fraction of edited alleles after 8 days of EGF deprivation (Fig. 4e). While initial prime editing efficiencies for most pegRNAs varied from 11% to 59%, we observed the enrichment of alleles harboring Ala289Asp/Thr/Val, Thr263Pro and all tested exon 20 insertions in the absence of EGF. Interestingly, the allelic fraction of the His773_Val774dup edit reached only 2.6% after selection in spite of an initial editing efficiency of 0.1%, thus representing a 23-fold enrichment and suggesting a strong phenotypic effect of this variant.

Next, we set out to evaluate the impact of additional synonymous variants on pegRNA enrichment and the sensitivity of our screen. We, thus, compared the enrichment of pegRNAs introducing only intended edits with that of constructs harboring additional synonymous mutations (Extended Data Fig. 10d). This revealed that the majority of hits were enriched with both vector types while showing similar or increased enrichment in the presence of synonymous edits. Surprisingly, Val774Met, Ser1026Gly and Gly804Gln were enriched only in the absence of synonymous edits, while Pro1090Leu and Gln581Leu/Lys were only enriched in their presence. Individual validation of these variants failed to demonstrate their enrichment in the absence of EGF, suggesting that they are false positives resulting from experimental noise (Extended Data Fig. 10e). Taken together, these results suggest that additional synonymous edits can serve to increase both prime editing efficiency and library redundancy, thus increasing the confidence in hits enriched in both their presence and their absence.

While many pathogenic mutations were identified as hits in our prime editing screen, future improvements to the prime editing technology will likely increase the sensitivity of the assay. Taken together, prime editing screens represent a promising avenue to expand genetic diversity in mutational scans and reveal pathogenic mutations undiscoverable by base editing, such as short insertions.

## Discussion

In this study, we developed complementary MAVEs using CBEs, ABEs and prime editing technology. We tested our approach by introducing tens of thousands of mutations in *EGFR* in multiple cell lines and in response to EGF deprivation or treatment with clinically approved TKIs gefitinib or osimertinib. EGF deprivation screens in wild-type *EGFR* cells revealed new activating variants not listed in ClinVar or classified as VUS that lead to EGF-independent growth, indicating that these may be bona fide oncogenic mutations. TKI treatment screens in wild-type and mutant *EGFR* cells uncovered a spectrum of variants leading to distinct profiles of drug resistance and susceptibility, suggesting that our approach may guide drug prioritization. EGFR variants were identified in the heavily studied tyrosine kinase domain and other clinically underappreciated domains, including the C-terminal tail. These results, thus, demonstrate the use of multimodal base editing and prime editing scans to assess variant pathogenicity and interrogate multiple mechanisms of oncogenic transformation and drug interactions.

Unlike conventional CRISPR screens, precision editing screens with base and prime editors increase our ability to link genotype to phenotype at single-nucleotide resolution. This is a critical step toward appreciating the tremendous genetic diversity identified in humans with disease and in particular conditions exemplified by genetic instability such as cancer. While prior base^31–34^ or prime^40^ editor screens could link genotype to phenotype in cellular assays, they were limited in terms of redundancy and genetic diversity or context. We demonstrate the utility of prime editing scanning with a library spanning a whole gene and introducing thousands of patient-derived variants, whereby we combine three precision editors (CBE, ABE and prime editor) to expand the number and diversity of genetic variants that can be introduced, as well as the coverage of patient-specific mutations that can be modeled at an endogenous target site.

One limitation of our screen is that it only considered EGF-independent proliferation as a proxy for EGFR signaling. In the future, these approaches can be easily applied to other cellular models to assess modes of EGFR dysregulation involving interactions with other receptors of the Erbb family or other ligands such as epiregulin^65^. Similarly, our pooled approach cannot distinguish between the effects of homozygous and heterozygous mutations, which might impact the signaling of oncogenic variants with different multimerization requirements^66^. However, these can be resolved as part of follow-up work in genetically engineered cell lines.

Surprisingly, we did not observe any overlap between prime editing and base editing screens, highlighting both the complementarity and the respective limitations of each technology. Because of the bystander effects of base editors, it is impossible to directly associate specific genotypes with a phenotype. To overcome this, we showed that direct sequencing of the endogenous target site could resolve the causal variant. In the future, this concept could be established in a screening context, such as through long-read sequencing, short-read sequencing of smaller coding segments, direct target capture, multiplexed inversion probes or a sensor library approach^38,67,68^. Nonetheless, it is worth noting that, even when known resistant variants cannot be modeled by base editors, our screens captured many critical protein residues and known intramolecular regulatory mechanisms. Prime editing technology currently suffers from unpredictable efficiency, which limits the sensitivity to implicate a broad spectrum of genetic mutations to phenotypes. This could likely be overcome in several ways, including expressing pegRNA-stabilizing proteins or increasing nucleotide availability in cells^69,70^, leveraging pegRNA efficiency prediction tools^71,72^ and sequencing the endogenous target sites rather than the pool of pegRNAs. One further improvement impacting both base and prime editing is spacer availability, which is the main factor preventing the modeling of all known ClinVar variants and limiting redundancy. In the future, this may be overcome through using alternative natural or engineered Cas variants with different PAM specificities or increasing the efficiency of longer-range prime edits. Lastly, pegRNAs introducing additional synonymous mutations have been shown to increase prime editing efficiency and our results demonstrate their usefulness to increase pegRNA library redundancy and confidence in identified hits.

In conclusion, we established a multimodal precision base editing and prime editing mutational scanning framework to interrogate genetic elements at high and single-nucleotide resolution. Our approach yielded fundamentally new insights into EGFR activation and resistance to clinically approved TKIs, opening avenues for further work that may one day guide clinical decision making. We anticipate that multimodal mutational scanning using precision editing can be expanded in the future to fully saturate genetic variant diversity in seemingly any genetic element, helping us to interpret the importance of yet-to-be-discovered genetic variants in a clinical context. We demonstrated the importance of deploying these approaches in diverse cellular contexts and expect that base editing and prime editing screens will be of particular interest in relevant models requiring DNA double-strand break-free editing, as recently demonstrated in primary human cells^73,74^.

## Methods

### Vectors

For BE3.9max and ABE8e experiments, individual spacers and libraries were cloned into the pRDA_256 (Addgene, 158581) and pRDA_426 (Addgene, 179097) plasmids, respectively.

For prime editing, a pLentiGuide vector (Addgene, 117986) was modified by replacing the existing BsmBI sites and the sequence in between by the following stuffer, thus changing the BsmBI overhangs (in bold): **CACC**GGAGACGCTATCACCCCGTCTCT**TTTT**. Additionally, the mCherry CDS was replaced by GFP and all pegRNAs were cloned in the resulting pLentiGuide_Puro-T2A-GFP vector.

The prime editor enzyme was delivered to cells by the pLenti-PE2max-BSD vector (Addgene, 191102).

### Cell lines and culture

PC-9 cells were obtained from Merck (90071810-1VL) and cultured in RPMI medium (ATCC modification; ThermoFisher, A1049101) supplemented with 10% FBS and 1% penicillin–streptomycin (ThermoFisher, 15140122).

MCF10A cells were obtained from the Cell Lines Service (305026) and cultured in complete mammary epithelial cell growth medium (MEGM bullet kit; Lonza, CC-3150) supplemented with 100 ng ml^−1^ cholera toxin (Merck, C8052). For EGF deprivation experiments, the same medium was used, omitting the hEGF provided with the bullet kit. Culture medium was replaced every 4 days.

For prime editing experiments, polyclonal MCF10A cells stably integrating the PEmax construct were derived using lentiviral delivery and regularly selected with 20 μg ml^−1^ blasticidin S.

PC-9 and MCF10A cell lines were cultured at 37 °C in 5.0% CO_2_ and passaged around 70% confluence. PC-9 cells were passaged using TrypLE (ThermoFisher, 12604013); MCF10A cells were passaged by washing with Dulbecco’s phosphate buffered saline (Merck, D8537) and using 0.05% trypsin-EDTA (ThermoFisher, 25300054). Trypsin was inhibited using soybean trypsin inhibitor (ThermoFisher, 17075029), as per the supplier’s instructions.

### Base editing library design

ChopChopv2 (https://chopchop.cbu.uib.no)^75^ was used to design all possible SpCas9 spacers targeting within and 30 nt around *EGFR* exons and UTRs (1,496 unique sgRNAs), as well as 200 unique sgRNAs targeting *EGFR* introns. sgRNAs containing BsmBI restriction sites or polyT(5) were filtered out and an additional G was appended to the 5′ end of spacers starting with another nucleotide.

Additionally, 206 (10% of the final library size) spacers with no target site in the human genome were picked at random from the Brunello library^76^. Lastly, 103 sgRNAs targeting splice sites of essential genes were obtained from Hanna et al.^33^ and added to the final library (Supplementary Tables 4 and 5).

Base editing outcomes were predicted for each *EGFR*-targeting sgRNA using the base editor design tool script from Hanna et al.^33^.

### Base editing library cloning

Primer-binding sites and BsmBI Golden Gate cloning sequences were added to the 5′ and 3′ ends of each spacer, respectively:

5′-AGGCACTTGCTCGTACGACG(**CGTCTC**)ACACC-3′,

5′-GTTTC(**GAGACG**)TTAAGGTGCCGGGCCCACAT-3′.

The final oligo library was ordered from Twist Bioscience and amplified in five replicates of the following reaction: 25 μl of Kapa Taq ReadyMix (Merck, TAQKB), 6 μl of primer mix (2.5 μM each), 1 μl of oligo pool (1 ng μl^−1^) and 17 μl of H_2_O. PCR cycling conditions were as follows: 3 min at 95 °C, followed by 20 s at 98 °C, 15 s at 60 °C and 30 s at 72 °C for 20 cycles, with a final extension for 1 min at 72 °C.

All PCR replicates were then pooled and desalted with a Qiagen PCR purification kit (Qiagen, 28104). The obtained double-stranded library inserts were then cloned into the ABE8e and BE3.9max lentiviral backbones using Golden Gate assembly: 50 fmol of amplified library, 50 fmol of plasmid backbone, 1 μl of ThermoFisher Tango buffer (ThermoFisher, BY5), 1 μl of DTT (10 mM), 1 μl of ATP (10 mM), 1 μl of T7 DNA ligase, 1 μl of BsmBI (Esp3I; ThermoFisher, ER0451). Thermocycler conditions were as follows: 5 min at 37 °C and 5 min at 20 °C for 25 cycles followed by 1 h at 37 °C and 10 min at 65 °C.

Golden Gate assembly products were then purified by isopropanol purification (as described by Joung et al.^77^) and 100 ng from each purified product was electroporated in each of three shots of Endura electrocompetent *Escherichia coli* (Lucigen, 60242) following the manufacturer’s protocol. The cells were then grown overnight in 500 ml of Terrific Broth supplemented with 100 mg L^−1^ ampicillin. Plasmid libraries were purified using the Qiagen plasmid maxiprep kit (Qiagen, 12162).

### Prime editing library design

All variants affecting *EGFR* exons and UTRs with a maximum size of 10 nt were obtained from the ClinVar (accessed in February 2024; 1,830 variants) and COSMIC (v99; 846 variants) databases and added to 1,208 variants predicted to be introduced in our base editing experiments, yielding a total of 3,191 unique variants. For each variant, pegRNAs were designed with PrimeDesign^78^ by allowing a maximum distance of 20 nt between the nicking site and the edit. Three versions of each pegRNA were designed with PBS lengths of 11, 13 or 15 nt. Whenever possible, additional synonymous mutations were added to a codon adjacent to the edit and/or within the PAM sequence to increase MMR escape and prevent edited site retargeting. In these cases, control pegRNAs introducing only the intended or the synonymous edit separately were added to the library to control for unexpected synonymous mutation effects. Lastly, 999 nontargeting pegRNAs were added to the library and constructs containing BsmBI restriction sites or polyT(5) were filtered out, yielding a final library size of 54,007 unique pegRNAs (Supplementary Table 6). The custom R scripts used for pegRNA library design are available from GitHub (https://github.com/plattlab/multimodal_genetic_variants).

For each library element, the pegRNA backbone was replaced with a pair of BsmBI sites separated with a random stuffer whose length was designed to normalize the final oligonucleotide length to 225 nt regardless of pegRNA extension design. The tevopreQ sequence was appended to the 3′ end of each pegRNA extension followed by a Pol III termination signal and a unique 11-nt DNA barcode. Lastly, final oligonucleotides were designed by adding the following 5′ and 3′ Gibson cloning overhangs:

5′-TATATATCTTGTGGAAAGGACGAAACACCG-3′,

5′-TTTTCGAGTACTAGGATCCATTAGGCG-3′.

### Prime editing library cloning

The final oligo library was ordered from Twist Bioscience and amplified in 65 replicates of the following reaction: 25 μl of Kapa Taq ReadyMix (Merck, TAQKB), 6 μl of primer mix (2.5 μM each), 1 μl of oligo pool (1 ng μl^−1^) and 17 μl of H_2_O. PCR cycling conditions were as follows: 3 min at 95 °C, followed by 20 s at 98 °C, 15 s at 61 °C and 30 s at 72 °C] for eight cycles, with a final extension for 1 min at 72 °C.

Library cloning was then performed in two steps. First, all PCR replicates were pooled and desalted with a Qiagen PCR purification kit (Qiagen, 28104). The obtained double-stranded library inserts were then cloned into the predigested pB693_pLentiGuide_Puro-T2A-GFP vector by Gibson assembly using the NEBuilder HiFi DNA assembly master mix (New England Biolabs, E2621).

Gibson assembly products were then purified by isopropanol purification (as described by Joung et al.^77^) and 100 ng from each purified product was electroporated in each of 8 shots of Endura electrocompetent *E.* *coli* cells (Lucigen, 60242) following the manufacturer’s protocol. The cells were then grown overnight in 500 ml of Terrific Broth supplemented with 100 mg L^−1^ ampicillin. The plasmid library was purified using the Qiagen plasmid maxiprep kit (Qiagen, 12162).

The sgRNA backbone was then inserted in the previously cloned plasmid library using Golden Gate assembly: 50 fmol of library plasmid, 50 fmol of sgRNA backbone oligo duplex, 1 μl of ThermoFisher Tango buffer (ThermoFisher, BY5), 1 μl of DTT (10 mM), 1 μl of ATP (10 mM), 1 μl of T7 DNA ligase and 1 μl of BsmBI (Esp3I; ThermoFisher, ER0451). Thermocycler conditions were as follows: 5 min at 37 °C and 5 min at 20 °C for 25 cycles followed by 1 h at 37 °C and 10 min at 65 °C. The final plasmid library was then purified, desalted and amplified following the previously described protocol.

### Lentivirus production

For each sgRNA library, HEK293T cells (Merck, 12022001) seeded in 15 (base editing) or 30 (prime editing) 15-cm dishes were transfected by preparing the following mixture for each dish: 5,548 ng of psPAX2 plasmid, 4,356 ng of pMD2 plasmid, 11,087 ng of library plasmid, 169 μl of PEImax (1 g L^−1^), 400 μl of NaCl (1.5 M) and H_2_O to 2,000 μl.

The transfection mixes were vortexed for 10 s, incubated at room temperature for 10 min and added dropwise to the cells.

Transfected cells were incubated for 48 h at 37 °C before viral supernatants were harvested, pooled and clarified 5 min at 1,000*g*. Lentiviral solutions were then overlaid on top of a 3-ml 20% sucrose cushion and centrifuged in a SW32Ti swing-bucket rotor (90 min at 25,000*g*, 4 °C). Lentiviral pellets were then resuspended in 1 ml of DMEM + 2% FBS and frozen at −80 °C.

### Screening and drug selection

For base editing screens, PC-9 or MCF10A cells were infected in duplicates while aiming for a coverage of 500 infected cells per library element and an MOI of 0.3. Infected cells were selected with 3 μg ml^−1^ puromycin from day 2 to day 4 and split into the different treatment arms on day 11. Final harvest was performed on day 19 while maintaining a minimal cell coverage of 500×.

For the prime editing screen, infection was performed with a cellular coverage of 250× and selection started on day 14. The final harvest was performed on day 22 by maintaining the 250× coverage for untreated samples while EGF-deprived samples showed coverages of 90× and 180× for replicates 1 and 2, respectively.

Cell pellets were frozen at −80 °C before processing. Each drug was used at a concentration corresponding to the half-maximal inhibitory concentration described in the literature or measured by cell counting after 4 days of selection. Specifically, PC-9 cells were treated with 0.05 μM gefitinib or 0.02 μM osimertinib. MCF10A cells were treated with 0.13 μM gefitinib or 0.3 μM osimertinib.

### Genomic DNA isolation and sequencing

Genomic DNA isolation was performed using the blood and cell culture DNA Midi kit (Qiagen, 13343) according to the manufacturer’s protocol.

Deep sequencing library preparation was performed in two steps. First, 4 μg of genomic DNA was used in each 50-μl PCR1 reaction with primers amplifying the sgRNA library and containing universal Illumina adaptors (Supplementary Table 7). Forward primers were ordered from Integrated DNA Technologies to include three different stagger lengths and were pooled in equal amounts. The final PCR mixes had the following composition: 25 μl of Kapa Taq ReadyMix (Merck, TAQKB), 6 μl of primer mix (2.5 μM each), 4 μg of genomic DNA and H_2_O to 50 μl. PCR cycling conditions were as follows: 3 min at 95 °C, followed by 20 s at 98 °C, 15 s at 60 °C and 30 s at 72 °C for 18 cycles, with a final extension for 30 s at 72 °C. PCR1 products from the same sample were then pooled together. In the case of prime editing screens, PCR1 amplicons were size-selected using a 1.3× Ampure bead ratio and eluted in a volume of H_2_O equal to the initial PCR product volume.

For each sample, a single PCR2 reaction was then performed to add P5 and P7 Illumina adaptors and demultiplexing barcodes. The reaction mix was as follows: 10 μl of Kapa Taq ReadyMix (Merck, TAQKB), 2.5 μl of primer mix (4 μM each), 1 μl of PCR1 product and 6.5 μl of H2O. PCR cycling conditions were as follows: 3 min at 95 °C, followed by 20 s at 98 °C, 15 s at 61 °C and 30 s at 72 °C for eight cycles, with a final extension for 30 s at 72 °C.

Barcoded PCR2 products were then pooled and desalted and 700 ng of DNA was loaded in each lane of a 2% agarose E-Gel (Invitrogen, A42135). DNA bands were extracted using the Qiagen PCR purification kit (Qiagen, 28104).

Samples were sequenced on a Illumina NextSeq 500 sequencer (SR150 HighOutput) with 5% PhiX.

### Base editing and prime editing screen data analysis

sgRNA or pegRNA barcode count tables were obtained using a custom Python script. Briefly, deep sequencing reads were trimmed with trimmomatic^79^ and sgRNA or barcode sequences were extracted by searching for adaptor sequences directly preceding and following them (Supplementary Table 7). One mismatch was allowed in each adaptor sequence but only perfect sgRNA or barcode matches were considered. LFCs and false discovery rate (FDR) values were then calculated with MAGeCK^43^ using default settings after normalizing read counts to nontargeting guides or pegRNAs. sgRNAs or barcodes with an average normalized read count lower than 50 reads in any treatment arm were ignored. Variant classifications were obtained from ClinVar (last accessed in February 2024) and COSMIC (version 99).

### Validation

Individual sgRNAs and pegRNAs were delivered to cells using the same lentiviral vector used for the screens (Supplementary Table 8). Infected cells were selected with puromycin from day 2 to day 4 after infection for base editing validations and day 2 to day 7 after infection for prime editing validations. For deep sequencing validation, drug treatments or EGF depletion were applied from day 6 to 13 for base editing experiments and day 7 to 15 for prime editing experiments before the final cell harvest.

Genomic DNA was extracted by resuspending cell pellets in QuickExtract buffer (Lucigen, QE09050) at a final concentration of 1,600 cells per μl and incubating them for 5 min at 65 °C, 5 min at 68 °C and 10 min at 98 °C. Then, 9.5 μl of genomic DNA (15,200 cells) was used for deep sequencing.

Sequencing data were analyzed with CRISPResso2 (ref. ^69^) using base editing quantification. For prime editing experiments, the HDR quantification mode was used by providing expected editing products. In all experiments, samples with fewer than 3,000 aligned reads were excluded from the analysis. CRISPResso parameters used for each experiment are available in the Source data.

For cell viability experiments, cells were seeded in 96-well plates (Greiner, 655077) 4 days after infection, treatments were applied on day 5 and viability measurements were performed on day 10 using the CellTiter-Glo 2.0 cell viability assay kit (Promega, G9241). Luminescence was measured following the manufacturer’s protocol on a Tecan SPARK reader.

### Endogenous *MLH1* gene knockout in MCF10A cells

MCF10A cells were seeded in a 24-well plate and transfected on the following day with a plasmid expressing the 5′-GAAGACAATGGCACCGGGATC-3′ sgRNA targeting the exon 2 of *MLH1*. Each well was transfected with the following mixtures: 1.5 μg of U6-sgRNA_CAG_SpCas9-2A-Puro plasmid, 10.8 μl of PEImax (1 g L^−1^), 24 μl of NaCl (1.5 M) and H_2_O to 120 μl. Clonal cell lines were then isolated after antibiotic selection and complete gene knockout was confirmed by Sanger sequencing of the genomic target and western blot.

### Figure preparation

All plots were generated using the GGplot (https://ggplot2.tidyverse.org) and GGally (https://ggobi.github.io/ggally/) packages in RStudio. Elements of Figs. 1a and 4b were drawn using icons from Marcel Tisch under a Creative Commons 1.0 Universal license (https://creativecommons.org/publicdomain/zero/1.0/). Elements of Figs. 1b and 2a were drawn using icons from Servier Medical Art, by Servier, under a Creative Commons 4.0 Attribution license (https://creativecommons.org/licenses/by/4.0/).

### Reporting summary

Further information on research design is available in the Nature Portfolio Reporting Summary linked to this article.

## Online content

Any methods, additional references, Nature Portfolio reporting summaries, source data, extended data, supplementary information, acknowledgements, peer review information; details of author contributions and competing interests; and statements of data and code availability are available at 10.1038/s41587-024-02439-1.

## Supplementary information

Supplementary Information Supplementary Tables 1–3.

Reporting Summary

Supplementary Tables 4–8 ABE8e sgRNA library. BE3.9Max sgRNA library. *EGFR*-targeting epegRNA library. Deep sequencing primers used in this study and adaptor sequences used for sgRNA and epegRNA library quantification. Individual sgRNAs and pegRNAs used in validation experiments.

## Source data

Source Data Fig. 1 Raw sgRNA counts and MAGeCK outputs for EGFR activation screens in MCF10A.

Source Data Fig. 2 Raw sgRNA counts and MAGeCK outputs for drug resistance screens in MCF10A.

Source Data Fig. 3 Raw sgRNA counts, MAGeCK outputs and CRISPResso2 parameters for drug resistance screens in PC-9 cells.

Source Data Fig. 4 Raw sgRNA counts, MAGeCK outputs and CRISPResso2 parameters for epegRNA EGFR activation screen in MCF10A cells.

## Data Availability

Genetic variant classification data used in this study were obtained from the ClinVar (https://www.ncbi.nlm.nih.gov/clinvar/, last accessed in February 2024) and COSMIC (https://cancer.sanger.ac.uk/cosmic, version 99, last accessed in February 2024) databases. Published protein structures used in this study are available from the PDB under accession codes 2ITY and 6JXT. Raw sequencing data generated for this study are available from the Sequence Read Archive (PRJNA1044808). Source data are provided with this paper.

## Code availability

R scripts used to design the pegRNA library and Python scripts used to extract sgRNA and barcode counts from deep sequencing data are available from the Platt lab GitHub (https://github.com/plattlab/multimodal_genetic_variants)^80^.
