# Supplementary material for: Multimodal scanning of genetic variants with base and prime editing
Source: Nat Biotechnol. 2024 Nov 12;43(9):1458–70. doi: 10.1038/s41587-024-02439-1 (PMC12440817; doi:10.1038/s41587-024-02439-1)
Supplement: Supplementary file 1 — Supplementary Tables 1–3. [file 41587_2024_2439_MOESM1_ESM.pdf]

nature biotechnology

Supplementary information https://doi.org/10.1038/s41587-024-02439-1

Multimodal scanning of genetic variants with base and prime editing

In the format provided by the authors and unedited

sgRNA sequence	Editor	Log-fold change	FDR	Predicted amino acid change	Database category	Database accession	Most prevalent tissue in COSMIC	Comments	References
GTCTGGAAGTACGCAGACGC	ABE8e	0.61674	1.35E-12	Lys609Gly	Not listed			Tyr270, Asp587 and Lys609 form hydrogen bonds contributing to the auto-inhibitory conformation of the inactive receptor.	48, 82
GGCCGACAGCTATGAGATGG		0.51856	5.33E-13	Asp314Gly, Ser315Gly	Uncertain significance (Ser315Gly)	VCV001036295.6, COSV51810209	Breast (Asp314Gly)	Asp314Gly was observed in breast carcinoma.	
GCTCCCAGTACCTGCTCAAC		0.44004	6.79E-07	Gln812Arg	Uncertain significance	VCV002418796.2		Observed in NSCLC patients.	83
GGTGTATAAGGTAAGGTCCC		0.41104	3.47E-10	Tyr727Cys, Lys728Glu	Uncertain significance (Tyr727Cys)	VCV001026153.5, COSV51766928	Lung (Tyr727Cys)	Observed in NSCLC patients. Tyr727 is a phosphorylation site of the tyrosine kinase domain.	47, 84, 85
GCGATCTCCACATCCTGCCGG		0.39385	1.23E-03	Asp368Gly	Not listed				
GTGGGGCCGACAGCTATGAGA		0.35574	2.66E-04	Asp314Gly, Ser315Gly	Uncertain significance (Ser315Gly)	VCV001036295.6, COSV51810209	Breast (Asp314Gly)	Asp314Gly was observed in breast carcinoma.	
GCTGAATGACAAGGTAGCGCT		0.34238	3.51E-03	Ile981Thr, Val980Ala	Uncertain significance (Ile981Thr)	VCV002105917.1			
GAAGATCAAAGTGCTGGGCTC		0.33496	6.16E-04	Ile715Val, Lys716Gly	Not listed			Lys716 is a ubiquitination site.	46
GCTGCTGAAGAAGCCCTGCTG		0.30384	4.20E-04	Phe1024Pro	Not listed				
GACCTGCCCGGCAGGAGTCA	BE3.9max	0.83855	3.13E-127	Thr594Thr, Cys595Cys, Pro596Ser	Pathogenic (Pro596Ser)	VCV002582280.1, COSV51816377	Central nervous system	Pro596Ser was observed in glioma patients. Pro596 is located in a loop stabilizing the interaction between Asp587 and Lys609 and contributing to receptor auto-inhibition.	48
GATCACGCAGCTCATGCCCTT		0.66193	2.27E-80	Thr790Met, Gln791Ter	Drug response (Thr790Met)	VCV000016613.27, COSV51765492	Lung (Thr790Met)	Thr790 is the gatekeeper residue of the tyrosine kinase ATP-binding site. Thr790Met is the most prevalent secondary mutation conferring resistance to first generation TKIs . It has also been shown to increase the EGFR kinase activity and to be oncogenic on its own.	86, 87
GCGTCAATGTAGTGGGCACAC		0.56653	1.94E-22	Asp587Asn	COSMIC	COSV51849240	Large intestine / Central nervous system	Observed in glioma patients. Tyr270, Asp587 and Lys609 form a salt bridge contributing to the auto-inhibitory conformation of the inactive receptor.	48, 82
GAGCTGTCGGCCCCACAGGCT		0.52444	9.07E-42	Asp314Asn (predicted) Glu317Lys (observed)	Uncertain significance (Asp314Asn) / COSMIC (Glu317Lys)	VCV001440221.4, COSV51765881	Central nervous system (Glu317Lys)	Glu317Lys was observed in glioma.	88
GGGCTCCGGTGCGTTCGGCA		0.49083	4.44E-45	Gly719Gly, Ser720Phe	COSMIC (Ser720Phe)	COSV51780206	Lung (Ser720Phe)	Ser720Phe was observed in NSCLC patients.	44, 45
GCATACCAGAGAGCTCAGGAG		0.45833	5.69E-33	Exon25:+1, Leu1038Leu	Not listed			Different EGFR C-terminal truncated variants have been observed in glioblastoma. EGFRΔEx26-28 has been shown to promote EGFR signalling and confer anchorage-independent growth in absence of EGF in NIH-3T3 cells.	50, 51, 53
GTCATACCAGAGAGCTCAGGA		0.3728	8.19E-16	Exon25:+1, Leu1038Leu	Not listed				
GCATCACGTAGGCTTCCTGGA		0.3705	3.44E-20	Val765Ile	Not listed			Located in the regulatory αC-helix. Val765-Met766>X is a similar TKI-resistant indel found in NSCLC.	89
GACCTGCCCGGCAGGAGTCAT		0.35593	9.81E-15	Cys595Cys, Pro596Leu	Uncertain significance (Pro596Leu)	VCV001058870.5	Central nervous system (Pro596Leu)	Pro596 is located in a loop stabilizing the interaction between Asp587 and Lys609 and contributing to receptor auto-inhibition. It was observed in glioma patients.	48, 63
GTCCACGCTGGCCATCACGT		0.33849	1.88E-13	Val769Ile, Ser768Asn	Uncertain significance (Ser768Asn)	VCV001015382.5, COSV51821681	Lung (Ser768Asn)	Located the in the loop following the regulatory αC-helix of the kinase domain. Ser768 phosphorylation by CAMK2 inhibits EGFR activity. Multiple Ser768 variants like Ser768Ile are known activate EGFR and confer drug resistance.	89, 90, 91

Supplementary table 1: Screen hits of the base editing EGFR activation screen.

sgRNA sequence	Editor	Log-fold change	FDR	Predicted amino acid change	Database category	Database accession	Most prevalent tissue in COSMIC	Comments	References
GTGGCCATCACGTAGGCTTCC	ABE8e	1.9922	0	Met766Thr	COSMIC	COSV51815137	Lung	Observed in NSCLC patients. Met766 is located in the regulatory αC-helix and is part of the Gefitinib binding pocket. Met766Thr was previously shown to confer Gefitinib resistance in vitro.	54, 92
GCATGTCAAGATCACAGATTT		1.5626	3.77E-107	Lys852Gly	Not listed			In the active conformation Lys852 is involved in an hydrogen-bond network with Gln791, Asp1012 and Asp1014. Destabilizing this network has been shown to reduce Osimertinib binding affinity.	55, 91
GCAAGATCACAGATTTTGGGC		1.4027	2.26E-39	Ile853Val, Thr854Ala	Uncertain significance (Thr854Ala)	VCV001394709.2, COSV51796944	Lung (Thr854Ala)	Thr854Ala was observed as an acquired resistant mutation in NSCLC patients treated with 1st generation TKIs. Thr854 is part of the Gefitinib binding pocket.	58, 92, 93
GATACACCGTGCCGAACGCAC		1.1084	3.24E-25	Val726Ala	Not listed			Val726Ala is not listed in databases. However, Val726Met was reported to be insensitive to Gefitinib in NSCLC patients. Val726 has been shown to be directly involved in Gefitinib binding to WT EGFR.	94, 95
GTCCACGCTGGCCATCACGT		1.0903	1.15E-42	Val769Ala	COSMIC	COSV51782791	Lung	Val769 is located in the loop following the regulatory αC helix of the kinase domain and frequently affected by pathogenic and drug-resistant insertions. A NSCLC patient with Val769Ala was reported to respond positively to Erlotinib treatment.	89, 96
GATGTCAAGATCACAGATTTT		1.018	3.47E-34	Lys852Gly	Not listed			In the active conformation Lys852 is involved in an hydrogen-bond network with Gln791, Asp1012 and Asp1014. Destabilizing this network has been shown to reduce Osimertinib binding affinity.	55, 91
GCATCACGTAGGCTTCCTGGA		0.96265	7.18E-26	Val765Ala	Not listed			Located in the regulatory αC-helix. Val765-Met766>X is a similar TKI-resistant indel found in NSCLC.	89
GACGTAGGCTTCCTGGAGGGA		0.75464	2.95E-17	Tyr764His	Not listed			Located in the regulatory αC-helix. Tyr764 is affected by the A763-Y764>FQEA insertion commonly found in NSCLC.	89
GATCACGCAGCTCATGCCCTT	BE3.9max	2.438	0	Thr790Met, Gln791Ter	Drug response (Thr790Met)	VCV000016613.27, COSV51765492	Lung (Thr790Met)	Thr790Met is the most prevalent TKI-resistant acquired mutation. It also has the strongest resistance to first generation TKI.	12
GTCCACGCTGGCCATCACGT		1.0931	1.14E-81	Val769Ile, Ser768Asn	Uncertain significance (Ser768Asn)	VCV001015382.5, COSV51821681	Lung (Ser768Asn)	Located the loop following the regulatory αC-helix of the kinase domain. Ser768 phosphorylation by CAMK2 inhibits EGFR activity. Multiple Ser768 variants are known activate EGFR and confer drug resistance. Ser768Ile in particular has been shown to be resistant to Gefitinib in vitro.	23, 89, 90, 91
GTGGCCATCACGTAGGCTTCC		0.9223	1.82E-53	Ala767Thr, Met766Ile, Val765Val	COSMIC (Met766Ile)	COSV51804547	Non specified	Met766Ile was observed in melanoma and is part of the Gefitinib binding pocket.	102. 97

Supplementary table 2: Screen hits of the base editing Gefitinib resistance screen in MCF10A cells.

sgRNA sequence	Editor	Log-fold change	FDR	Predicted amino acid change	Database category	Database accession	Most prevalent tissue in COSMIC	Comments	References
GTGTTTTCACCAGTACGTTCC		2.3083	0	Val845Ala	COSMIC	COSV51835641	Lung	Val845Ala is not listed in ClinVar but the similar Val845Leu variant has conflicting reports of pathogenicity in the database.	
GCATGTCAAGATCACAGATTT		1.938	6.66E-228	Lys852Gly	Not listed			In the active conformation Lys852 is involved in an hydrogen-bond network with Gln791, Asp1012 and Asp1014. Destabilizing this network has been shown to reduce Osimertinib binding affinity.	55, 91
GATCACGCAGCTCATGCCCTT		1.6989	1.48E-290	Thr790Ala, Gln791Arg	COSMIC (both)	COSV51773932, COSM9583352	Oesophagus (Thr790Ala), Lung (Gln791Arg)	Both mutations were observed in cancer patients. In the active receptor, Gln791 interacts with Lys852, Asp1012 and Asp1014. Destabilizing this network has been predicted to reduce Osimertinib binding affinity.	55, 91, 98, 99
GACAATCATCTGGCAGCGAGG		1.4736	1.94E-95	Alternative transcript					
GTGAATGACAAGGTAGCGCTG		1.1473	1.45E-42	Ile981Thr, Val980Ala	Uncertain significance (Ile981Thr)	VCV002105917.1		Observed in lung cancer.	
GTGCGTCTATCATCCAGCCTG		1.1367	7.90E-72	Ile953Thr	Uncertain significance	VCV000965600.6		Observed in lung cancer.	
GTCATATGGCTTGGATCCAA		1.0452	1.49E-30	Tyr915His	COSMIC	COSV51842912	Haematopoietic and lymphoid	Tyr915 is a phosphorylation site recognized by the c-Src tyrosine kinase.	100
GATGTCAAGATCACAGATTTT	ABE8e	1.0052	1.77E-33	Lys852Gly	Not listed			In the active conformation Lys852 is involved in an hydrogen-bond network with Gln791, Asp1012 and Asp1014. Destabilizing this network has been shown to reduce Osimertinib binding affinity.	55, 91
GAATGACAAGGTAGCGCTGG		0.98683	7.09E-30	Val980Ala, Leu979Leu	Not listed				
GAAGGTAGCGCTGGGGGTCTC		0.98396	3.91E-49	Tyr978His	Uncertain significance	VCV001386901.2		Y978 is phosphorylated in response to EGFR activation and subsequently recruits STAT5.	101
GAGCTGCGTGATGAGCTGCA		0.9828	2.98E-25	Ile789Thr, Leu792Pro	COSMIC (Leu792Pro)	COSV51860329	Lung	Leu792 is located in the ATP binding pocket of the kinase domain, in contact with bound Osimertinib. Leu792 variants have been found in patients with acquired Osimertinib resistance. Leu792His/Phe/Tyr have been shown to be resistant to Osimertinib in vitro.	102, 103
GCTGAATGACAAGGTAGCGCT		0.95711	4.66E-21	Ile981Thr, Val980Ala	Uncertain significance (Ile981Thr)	VCV002105917.1			
GCGATACAGCTCAGACCCCAC		0.93918	7.39E-19	Tyr1069Cys, Ser1070Gly	COSMIC (Tyr1069Cys)	COSV51765999	Large intestine / Biliary tract	Y1069 phosphorylation recruits the c-Cbl E3 ubiquitin ligase. Tyr1069Cys was shown to increases EGFR signaling and promotes EGF-independent cell growth in vitro.	104, 105
GTAGGAAATTTTAAAAGATGA		0.88042	2.66E-10	3'UTR					
GCAAGAAGATGCACGAAGGC		0.86791	2.42E-12	3'UTR					
GTGAGGCAGATGCCCAGCAGG		1.1808	1.92E-54	Cys781Tyr	Not listed			Cys781 is a palmitoylation site involved in EGFR localization at the plasma membrane.	106
GTTCTCCTTTCTCCAGGATGG		0.96163	4.43E-21	Gly930Lys	Not listed				
GCTTCTTCATCCATCAGGGCA	BE3.9max	0.82936	2.67E-21	Glu1005Lys, Glu1004Lys	Uncertain significance (Glu1004Lys)	VCV001385349.4, COSV51836372	Skin (Glu1004Lys)	Glu1005 directly interacts with Lys852 in the inactive conformation. It is part of a C-terminal "electrostatic hook" that inhibits the kinase domain activity. Mutating Glu1005 and Asp1006 has been shown to increase the activity of unstimulated EGFR in vitro.	56
GCGCTCACACCGTGCGGGGGG		0.80013	7.34E-14	5'UTR					

Supplementary table 3: Screen hits of the base editing Osimertinib resistance screen in MCF10A cells.

82. Ferguson, K. M. *et al.* EGF Activates Its Receptor by Removing Interactions that Autoinhibit Ectodomain Dimerization. *Molecular Cell* **11**, 507–517 (2003).

83. Koyama, N. *et al.* The Characterization of Gefitinib Sensitivity and Adverse Events in Patients with Non-small Cell Lung Cancer. *Anticancer Research* **26**, 4519–4525 (2006).

84. Gow, C.-H. *et al.* Comparison of epidermal growth factor receptor mutations between primary and corresponding metastatic tumors in tyrosine kinase inhibitor-naive non-small-cell lung cancer. *Annals of Oncology* **20**, 696–702 (2009).

85. Stabile, L. P. *et al.* Combined Targeting of the Estrogen Receptor and the Epidermal Growth Factor Receptor in Non–Small Cell Lung Cancer Shows Enhanced Antiproliferative Effects. *Cancer Research* **65**, 1459–1470 (2005).

86. Vikis, H. *et al.* EGFR-T790M Is a Rare Lung Cancer Susceptibility Allele with Enhanced Kinase Activity. *Cancer Research* **67**, 4665–4670 (2007).

87. Regales, L. *et al.* Development of New Mouse Lung Tumor Models Expressing EGFR T790M Mutants Associated with Clinical Resistance to Kinase Inhibitors. *PLOS ONE* **2**, e810 (2007).

88. Li, Y. Y. *et al.* Exome and genome sequencing of nasopharynx cancer identifies NF-κB pathway activating mutations. *Nat Commun* **8**, 14121 (2017).

89. Vyse, S. & Huang, P. H. Targeting EGFR exon 20 insertion mutations in non-small cell lung cancer. *Sig Transduct Target Ther* **4**, 1–10 (2019).

90. Huang, L.-C. *et al.* Integrative annotation and knowledge discovery of kinase post-translational modifications and cancer-associated mutations through federated protein ontologies and resources. *Sci Rep* **8**, 6518 (2018).

91. Mirza, A., Mustafa, M., Talevich, E. & Kannan, N. Co-Conserved Features Associated with cis Regulation of ErbB Tyrosine Kinases. *PLOS ONE* **5**, e14310 (2010).

92. Verma, N. *et al.* Identification of gefitinib off-targets using a structure-based systems biology approach; their validation with reverse docking and retrospective data mining. *Sci Rep*

6, 33949 (2016).

93. Zhang, L. *et al.* Molecular Characteristics of the Uncommon EGFR Exon 21 T854A Mutation and Response to Osimertinib in Patients With Non-Small Cell Lung Cancer. *Clinical Lung Cancer* **23**, 311–319 (2022).

94. Pallis, A. G. *et al.* ‘Classical’ but not ‘other’ mutations of EGFR kinase domain are associated with clinical outcome in gefitinib-treated patients with non-small cell lung cancer. *Br J Cancer* **97**, 1560–1566 (2007).

95. Todsaporn, D., Mahalapbutr, P., Poo-arporn, R. P., Choowongkomon, K. & Rungrotmongkol, T. Structural dynamics and kinase inhibitory activity of three generations of tyrosine kinase inhibitors against wild-type, L858R/T790M, and L858R/T790M/C797S forms of EGFR. *Computers in Biology and Medicine* **147**, 105787 (2022).

96. Xing, K. *et al.* A novel point mutation in exon 20 of EGFR showed sensitivity to erlotinib. *Med Oncol* **31**, 36 (2014).

97. Siroy, A. E. *et al.* Beyond BRAFV600: Clinical Mutation Panel Testing by Next-Generation Sequencing in Advanced Melanoma. *Journal of Investigative Dermatology* **135**, 508–515 (2015).

98. Sano, A. *et al.* Expression of receptor tyrosine kinases in esophageal carcinosarcoma. *Oncology Reports* **29**, 2119–2126 (2013).

99. Zhuo, M. *et al.* The Prognostic and Therapeutic Role of Genomic Subtyping by Sequencing Tumor or Cell-Free DNA in Pulmonary Large-Cell Neuroendocrine Carcinoma. *Clinical Cancer Research* **26**, 892–901 (2020).

100. Stover, D. R., Becker, M., Liebetanz, J. & Lydon, N. B. Src Phosphorylation of the Epidermal Growth Factor Receptor at Novel Sites Mediates Receptor Interaction with Src and P85α(*). *Journal of Biological Chemistry* **270**, 15591–15597 (1995).

101. Schulze, W. X., Deng, L. & Mann, M. Phosphotyrosine interactome of the ErbB-receptor kinase family. *Molecular Systems Biology* **1**, 2005.0008 (2005).

102. Chen, K. *et al.* Novel Mutations on EGFR Leu792 Potentially Correlate to Acquired Resistance to Osimertinib in Advanced NSCLC. *Journal of Thoracic Oncology* **12**, e65–e68 (2017).

103. Yang, Z. *et al.* Investigating Novel Resistance Mechanisms to Third-Generation EGFR Tyrosine Kinase Inhibitor Osimertinib in Non–Small Cell Lung Cancer Patients. *Clinical Cancer Research* **24**, 3097–3107 (2018).

104. Grøvdal, L. M., Stang, E., Sorkin, A. & Madshus, I. H. Direct interaction of Cbl with pTyr 1045 of the EGF receptor (EGFR) is required to sort the EGFR to lysosomes for degradation. *Experimental Cell Research* **300**, 388–395 (2004).

105. Koivu, M. K. A. *et al.* Identification of Predictive ERBB Mutations by Leveraging Publicly Available Cell Line Databases. *Molecular Cancer Therapeutics* **20**, 564–576 (2021).

106. Guo, H. *et al.* Targeting EGFR-dependent tumors by disrupting an ARF6-mediated sorting system. *Nat Commun* **13**, 6004 (2022).
