# Supplementary material for: Multimodal scanning of genetic variants with base and prime editing
Source: Nat Biotechnol. 2024 Nov 12;43(9):1458–70. doi: 10.1038/s41587-024-02439-1 (PMC12440817; doi:10.1038/s41587-024-02439-1)
Supplement: Supplementary file 2 — Reporting Summary [file 41587_2024_2439_MOESM2_ESM.pdf]

nature portfolio

Corresponding author(s): Randall J. Platt

Last updated by author(s): Sep 10, 2024

Reporting Summary

Nature Portfolio wishes to improve the reproducibility of the work that we publish. This form provides structure for consistency and transparency in reporting. For further information on Nature Portfolio policies, see our Editorial Policies and the Editorial Policy Checklist.

Statistics

For all statistical analyses, confirm that the following items are present in the figure legend, table legend, main text, or Methods section.

n/a	Confirmed	
☐	☒	The exact sample size (*n*) for each experimental group/condition, given as a discrete number and unit of measurement
☐	☒	A statement on whether measurements were taken from distinct samples or whether the same sample was measured repeatedly
☐	☒	The statistical test(s) used AND whether they are one- or two-sided<br>*Only common tests should be described solely by name; describe more complex techniques in the Methods section.*
☐	☒	A description of all covariates tested
☐	☒	A description of any assumptions or corrections, such as tests of normality and adjustment for multiple comparisons
☐	☒	A full description of the statistical parameters including central tendency (e.g. means) or other basic estimates (e.g. regression coefficient) AND variation (e.g. standard deviation) or associated estimates of uncertainty (e.g. confidence intervals)
☐	☒	For null hypothesis testing, the test statistic (e.g. F, t, r) with confidence intervals, effect sizes, degrees of freedom and P value noted<br>*Give P values as exact values whenever suitable.*
☒	☐	For Bayesian analysis, information on the choice of priors and Markov chain Monte Carlo settings
☒	☐	For hierarchical and complex designs, identification of the appropriate level for tests and full reporting of outcomes
☐	☒	Estimates of effect sizes (e.g. Cohen's *d*, Pearson's *r*), indicating how they were calculated

Our web collection on statistics for biologists contains articles on many of the points above.

Software and code

Policy information about availability of computer code

Data collection	Sequencing data were collected using an Illumina Nextseq 500 instrument and processed using publicly available softwares, as referenced in the methods section. sgRNA and barcode counts were extracted from sequencing data using a custom Python script that will be made available on the Platt Lab GitHub (https://github.com/plattlab/).<br>Trimmomatic 0.39 was used for Illumina read pre-processing.
Data analysis	The following publicly available softwares were used:<br>MAGeCK 0.5.9.2<br>CRISPResso 2.0.42<br>ChimeraX-1.8<br>R version 4.3.1 (2023-06-16)<br>ggplot2_3.4.4<br>GGally_2.1.2<br>Python 3.6.13<br>PrimeDesign (version 1, 2021-09-01)<br>CHOPCHOP (version 2)

For manuscripts utilizing custom algorithms or software that are central to the research but not yet described in published literature, software must be made available to editors and reviewers. We strongly encourage code deposition in a community repository (e.g. GitHub). See the Nature Portfolio guidelines for submitting code & software for further information.

Data

Policy information about availability of data

All manuscripts must include a data availability statement. This statement should provide the following information, where applicable:

- Accession codes, unique identifiers, or web links for publicly available datasets
- A description of any restrictions on data availability
- For clinical datasets or third party data, please ensure that the statement adheres to our policy

Genetic variant classification data used in this study were obtained from the ClinVar (https://www.ncbi.nlm.nih.gov/clinvar/, last accessed February 2024) and COSMIC (https://cancer.sanger.ac.uk/cosmic, v99) databases. Published protein structures used in this study are available from the Protein Data Bank under accession codes 2ITY and 6JXT. Raw sequencing data generated for this study are available at the Sequence Read Archive (PRJNA1044808). All unnormalized sgRNA and barcode counts are available in the Source Data section.

Human research participants

Policy information about studies involving human research participants and Sex and Gender in Research.

Reporting on sex and gender	n/a
Population characteristics	n/a
Recruitment	n/a
Ethics oversight	n/a

Note that full information on the approval of the study protocol must also be provided in the manuscript.

Field-specific reporting

Please select the one below that is the best fit for your research. If you are not sure, read the appropriate sections before making your selection.

☒ Life sciences ☐ Behavioural & social sciences ☐ Ecological, evolutionary & environmental sciences

For a reference copy of the document with all sections, see nature.com/documents/nr-reporting-summary-flat.pdf

Life sciences study design

All studies must disclose on these points even when the disclosure is negative.

Sample size	No statistical method was used to determine sample size. All screening experiments were conducted in duplicates which is standard in the field. Screening experiments were conducted with a coverage of at least 250 cells per library element to ensure that all sgRNAs/pegRNAs are represented in the transduced cell population. In each experiment, we quantified library distributions at early time points to confirm the absence of significant drop-outs and high sgRNA/pegRNA barcode count correlations across replicates.
Data exclusions	For all screening experiments, sgRNAs or barcoded epegRNAs with a normalized read count lower that 50 in any sample were excluded from the analysis. For validation experiments, samples with less than 3000 aligned reads to the reference amplicon were excluded.
Replication	High replicate correlation was measured and reported for all screening experiments. Screen hits were validated by delivering individual sgRNAs or pegRNAs to cells in three biological replicates and measuring editing efficiencies by deep sequencing. All attempts at replication were successful and the consistency of our screening approach was confirmed across screening conditions and cell lines.
Randomization	Randomization is not possible in pooled in vitro screens as cells are generally infected and divided into treatment groups from a common parent cell population.
Blinding	Investigators were not blinded for practical reasons as cell culture experiments were performed by a single investigator and morphological differences between treated and non-treated were visually obvious.

Reporting for specific materials, systems and methods

We require information from authors about some types of materials, experimental systems and methods used in many studies. Here, indicate whether each material, system or method listed is relevant to your study. If you are not sure if a list item applies to your research, read the appropriate section before selecting a response.

Materials & experimental systems

n/a	Involved in the study
☒	☐ Antibodies
☐	☒ Eukaryotic cell lines
☒	☐ Palaeontology and archaeology
☒	☐ Animals and other organisms
☒	☐ Clinical data
☒	☐ Dual use research of concern

Methods

n/a	Involved in the study
☒	☐ ChIP-seq
☒	☐ Flow cytometry
☒	☐ MRI-based neuroimaging

Eukaryotic cell lines

Policy information about cell lines and Sex and Gender in Research

Cell line source(s)	HEK293T cells were acquired from Sigma-Aldrich, PC-9 cells were obtained from Merck and MCF10A cells were obtained from Cell Lines Service.
Authentication	HEK293T, PC-9 and MCF10A cell lines were authenticated by their respective vendors using PCR amplification and analysis of short tandem repeat profiles.
Mycoplasma contamination	All cell lines were tested for mycoplasma contamination once a year and tested negative throughout the study.
Commonly misidentified lines (See ICLAC register)	No misidentified cell line was used.
